# Altered coronary artery function, arteriogenesis and endothelial YAP signaling in postnatal hypertrophic cardiomyopathy

**DOI:** 10.3389/fphys.2023.1136852

**Published:** 2023-03-31

**Authors:** Paulina Langa, Richard J. Marszalek, Chad M. Warren, Shamim K. Chowdhury, Monika Halas, Ashley Batra, Koreena Rafael-Clyke, Angelie Bacon, Paul H. Goldspink, R. John Solaro, Beata M. Wolska

**Affiliations:** ^1^ Department of Physiology and Biophysics, College of Medicine, University of Illinois at Chicago, Chicago, IL, United States; ^2^ Center for Cardiovascular Research, College of Medicine, University of Illinois at Chicago, Chicago, IL, United States; ^3^ Department of Medicine, Division of Cardiology, College of Medicine, University of Illinois at Chicago, Chicago, IL, United States

**Keywords:** hypertrophic cardiomyopathy, YAP signaling, mechano-signaling, coronary flow, echocardiography, fibrosis

## Abstract

**Introduction:** Hypertrophic cardiomyopathy (HCM) is a cardiovascular genetic disease caused largely by sarcomere protein mutations. Gaps in our understanding exist as to how maladaptive sarcomeric biophysical signals are transduced to intra- and extracellular compartments leading to HCM progression. To investigate early HCM progression, we focused on the onset of myofilament dysfunction during neonatal development and examined cardiac dynamics, coronary vascular structure and function, and mechano-transduction signaling in mice harboring a thin-filament HCM mutation.

**Methods:** We studied postnatal days 7–28 (P7–P28) in transgenic (TG) TG-cTnT-R92Q and non-transgenic (NTG) mice using skinned fiber mechanics, echocardiography, biochemistry, histology, and immunohistochemistry.

**Results:** At P7, skinned myofiber bundles exhibited an increased Ca^2+^-sensitivity (pCa_50_ TG: 5.97 ± 0.04, NTG: 5.84 ± 0.01) resulting from cTnT-R92Q expression on a background of slow skeletal (fetal) troponin I and α/β myosin heavy chain isoform expression. Despite the transition to adult isoform expressions between P7–P14, the increased Ca^2+^- sensitivity persisted through P28 with no apparent differences in gross morphology among TG and NTG hearts. At P7 significant diastolic dysfunction was accompanied by coronary flow perturbation (mean diastolic velocity, TG: 222.5 ± 18.81 mm/s, NTG: 338.7 ± 28.07 mm/s) along with localized fibrosis (TG: 4.36% ± 0.44%, NTG: 2.53% ± 0.47%). Increased phosphorylation of phospholamban (PLN) was also evident indicating abnormalities in Ca^2+^ homeostasis. By P14 there was a decline in arteriolar cross-sectional area along with an expansion of fibrosis (TG: 9.72% ± 0.73%, NTG: 2.72% ± 0.2%). In comparing mechano-transduction signaling in the coronary arteries, we uncovered an increase in endothelial YAP expression with a decrease in its nuclear to cytosolic ratio at P14 in TG hearts, which was reversed by P28.

**Conclusion:** We conclude that those early mechanisms that presage hypertrophic remodeling in HCM include defective biophysical signals within the sarcomere that drive diastolic dysfunction, impacting coronary flow dynamics, defective arteriogenesis and fibrosis. Changes in mechano-transduction signaling between the different cellular compartments contribute to the pathogenesis of HCM.

## 1 Introduction

Hypertrophic cardiomyopathy (HCM) is an inherited cardiac disorder occurring between 1 in 200 to 1 in 500 individuals (Semsarian et al., 2015). Mutations are linked to genes of the sarcomere or cytoskeletal network (Yotti et al., 2019). Although awareness has increased due to sudden cardiac death in young athletes, HCM more commonly presents with advanced age. HCM progresses insidiously from a prolonged stage of mild diastolic dysfunction (carrier state) to one of progressive cardiac remodeling, taking the form of myocardial hypertrophy and fibrosis with deterioration of cardiac function (Maron, 2018). The chronic progression in HCM complicates investigations into its pathophysiology by obscuring critical precursor events. Therefore, clinical presentation often encompasses relatively advanced disease on which most studies have focused.

We (Pena et al., 2010; Alves et al., 2014; Wilder et al., 2015; Ryba et al., 2019; Chowdhury et al., 2020) and others (Li et al., 2010) reported that early interventions reducing hypertrophic signaling can reduce or stop the progression of diastolic dysfunction, maladaptive remodeling, and fibrosis. There is conflicting evidence regarding the success of gene therapy to prevent HCM. Mearini et al. (2014) reported that myosin binding protein C (MyBP-C) gene therapy prevented HCM progression. However, Cannon et al. (2015) employing a common myosin heavy chain (MHC) mutation (Arg403Gln) linked to HCM reported that using a genetic approach to remove the mutant protein could not stop early progression of HCM progression. These investigators emphasized the importance of examining early non-sarcomeric maladaptive signaling in understanding and treating HCM. Accordingly, treatments with Ca^2+^ channel blockers or angiotensin receptor blockers (ARBs) for HCM proved ineffective after disease progression (Araujo et al., 2005; Flenner et al., 2017). In addition, mavacamten an allosteric inhibitor of cardiac myosin ATPase was only approved in patients with obstructive HCM (Keam, 2022). Moreover, it is unknown whether mavacamten can delay or stop progression in younger patients. Early treatment with the ARB, valsartan, has shown promise in diminishing diastolic dysfunction, but vascular mechanisms likely to be involved have not been thoroughly described (Ho et al., 2021).

Increases in myofilament response to Ca^2+^ are archetypal of HCM-linked thin filament mutations and are directly associated with delayed relaxation during diastole in HCM (Nagueh et al., 2001; Poutanen et al., 2006; Gandjbakhch et al., 2010). The initial diastolic dysfunction in HCM carriers, although not enough to impact ventricular filling, likely affects coronary flow dynamics, serving as a catalyst for progressive vascular alterations. Vascular remodeling is well-documented in human autopsy of HCM hearts, often leading to occlusions and micro-infarctions (Foa et al., 2019), but its early presence and impact remain unknown. Moreover, the contribution of non-myocyte cellular dysfunction in HCM has been gaining recognition, particularly with respect to micro-vessel dysfunction and ischemia (Maron et al., 2019). Yet, there remains a need for an in-depth understanding of the relationship between vascular function and remodeling in early HCM disease progression.

Results presented here provide evidence of an early decline in coronary flow velocity and changes in the vascular architecture, acting as likely triggers concomitant with the onset of expression of mutant cardiac troponin T (cTnT) at position 92 (R92Q) and mild diastolic dysfunction. We have also discovered temporal and spatial changes in novel signaling pathways in the initial phases of HCM development not previously considered. These changes precede fibrosis and atrial remodeling in the pathophysiology of HCM.

## 2 Methods

Expanded materials and methods can be found in the Supplementary Material.

### 2.1 Institutional approval and animal model

Experiments were approved by the Animal Care and Use Committee of the University of Illinois at Chicago and in compliance with the Guide for the Care and Use of Laboratory Animals Eighth Edition as adopted by the U.S. National Institutes of Health. Experiments employed male and female postnatal days 2 (P2), 4 (P4), 7 (P7), 10 (P10), 14 (P14), 21 (P21), and 28 (P28) non-transgenic (NTG) mice and transgenic (TG) mice expressing an HCM-linked cTnT-R92Q mutant generated on the FVB/N background strain as previously described (Chowdhury et al., 2020).

### 2.2 Skinned fiber bundles tension measurement

Hearts were excised and detergent-extracted (skinned) fiber bundles were prepared for force-Ca^2+^ measurements as described previously (Alves et al., 2014).

### 2.3 SDS-PAGE and immunoblotting

Whole-heart homogenates and isolated myofibrils from frozen hearts were prepared with a Bead Ruptor 24 Elite as outlined in the Supplementary Material. To separate isoforms of TnT and myosin heavy chain (MHC), 8% and 6% SDS-PAGE respectively was utilized (Anderson et al., 1991; Warren and Greaser, 2003). All other gels were 12% or 15% SDS-PAGE as described in the Supplementary Material. The abundance levels of phospho-modified and total unmodified PLN, CAMKII, ERK1/2, YAP and PKA-C as well as the total unmodified forms of eNOS, gp91phox (NOX2), NOX4, SERCA2a, calcineurin, (see Supplementary Table S4 for details) were analyzed with Image Lab v6.0.1 (BioRad).

### 2.4 Echocardiography

Transthoracic echocardiography was performed using a Vevo 2100 High-Resolution *In Vivo* Imaging System (FUJIFILM VisualSonics, 2100) as previously described (Alves et al., 2014; Chowdhury et al., 2020). In addition, left coronary flow measurements were performed subsequent to morphometric and functional cardiac assessment as described by Chang et al. (2015). All measurements and calculations were averaged from three consecutive cycles and performed according to the American Society of Echocardiography guidelines. Data analysis was performed with the VevoLab 5.5.1. Analytic Software.

### 2.5 Histology

The heart samples were fixed in 10% formalin, followed by paraffin embedding. Formalin-Fixed Paraffin-Embedded (FFPE) slides were de-paraffinized, rehydrated, and antigen-retrieved (Tris-EDTA) followed by blocking (5% BSA) (Chowdhury et al., 2020).

To assess fibrosis, we used slides stained with a Trichrome Stain kit (Abcam, cat. Ab150686) for visualization of collagenous fibrotic tissue. Pixels corresponding to the area stained in blue, indicating collagenous areas reflecting fibrosis, were normalized to the total pixel area of the tissue in the assessed image. Images of whole heart sections were taken by Zeiss Axio Imager Z2 (Germany) brightfield microscope.

Slides were incubated in anti-CD31, anti-αSMA, and anti-YAP antibodies for immunohistochemistry. After washing with PBS, they were incubated with secondary antibodies, followed by DAPI for nuclear counterstaining. Images were acquired with the Zeiss confocal LSM880 (Germany) microscope equipped with a motorized stage (see the Supplementary Material for details).

### 2.6 Statistical analysis

Gaussian distribution was assessed by the Shapiro-Wilk test and the homogeneity of variance was assessed by Brown-Forsythe or Levene’s test. Depending on the conditions, a two-way ANOVA followed by Fisher’s least significant difference (LSD), one-way ANOVA followed by Šidák correction, or unpaired two-tailed Student’s t-test were performed using GraphPad Prism v. 9.3.1. Detailed information on performed statistical tests is presented in the Figure legends. Significance was set to *p* < 0.05.

## 3 Results

### 3.1 Altered myofilament properties and changes in myofilament isoforms during increased expression of cTnT-R92Q

**FIGURE 1:**
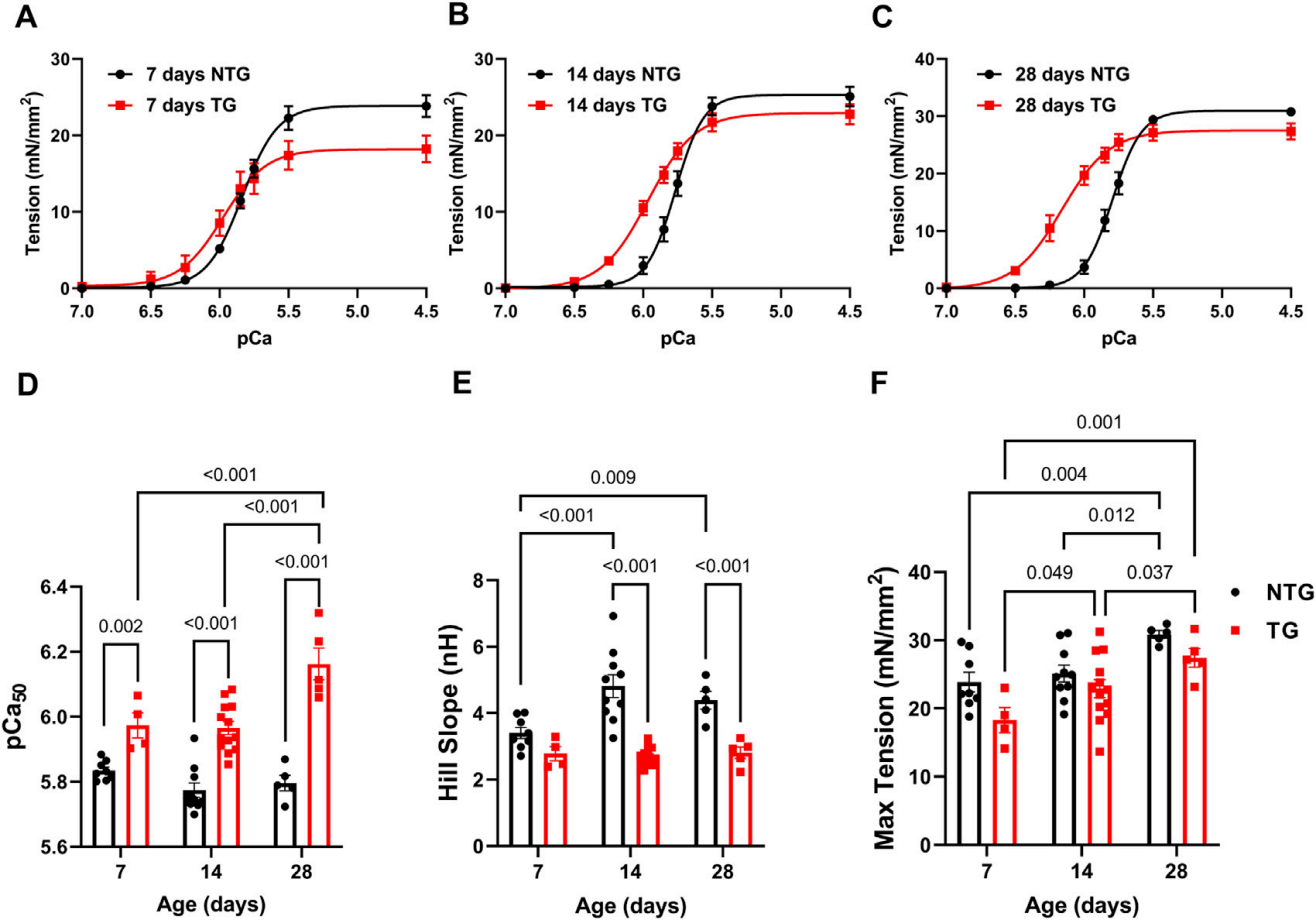
Age-dependent myofilament Ca^2+^ response. **(A)** Force-Ca^2+^ relation at P7, **(B)** at P14, and **(C)** at P28. **(D)** Summary of myofilament Ca^2+^ sensitivity (pCa_50_), **(E)** Hill slope, and **(F)** maximum tension. Data are presented as mean ± SEM. Data were analyzed by 2-way ANOVA followed by Fisher’s LSD test; N = 4–13. NTG, non-transgenic; TG, transgenic.

**FIGURE 2:**
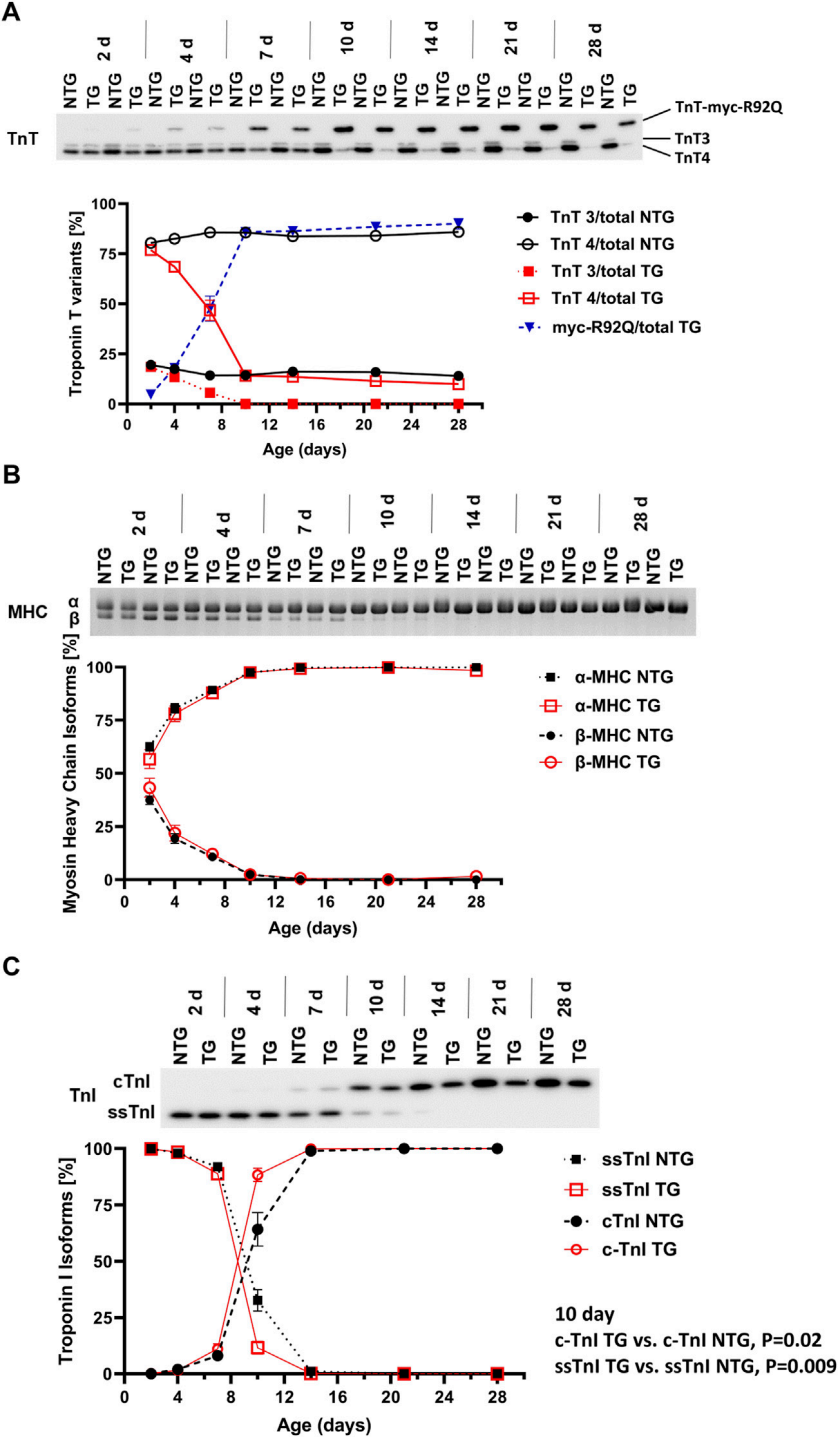
Timeline of myofilament isoform switches. Representative images of Western blots and SDS-PAGE summary data of expression of **(A)** troponin T (TnT) isoforms (N = 2 for both groups), **(B)** myosin heavy chain (MHC) isoforms, and **(C)** troponin I (TnI) isoforms in NTG and TG hearts from P2 to P28. For **(B,C)** N = 4 for both groups. Data are presented as mean ± SEM. NTG, non-transgenic; TG, transgenic. TnT3—adult splicing form 3 of Troponin T; TnT4—adult splicing form 4 of Troponin T; myc-R92Q, myc tag for TnT-R92Q mutation; ssTnI, slow skeletal isoform of TnI; cTnI, cardiac isoform of TnI.

Our previous studies with adult TG hearts expressing cTnT-R92Q showed that deletion of phospholamban (PLN) resulted in the correction of diastolic and systolic dysfunction as well as morphological and fibrotic maladaptation despite a persistently elevated myofilament Ca^2+^ sensitivity (Chowdhury et al., 2020). These findings emphasize the relation between the maintenance of myofilament Ca^2+^ response and early maladaptive changes in HCM. In view of the documented changes in the myofilament response to Ca^2+^ and myofilament protein isoform composition in immature sarcomeres (Karbassi et al., 2020), we first compared pCa-tension relations (Figure 1 and Supplementary Table S1) at P7, P14, and P28. We also assessed the expression of TnT, TnI, and MHC isoforms, and the expression of cTnT-R92Q at P2 to P28 (Figure 2).

At P7 TG-skinned fibers showed a significant increase in pCa_50_ [5.97 ± 0.04 (N = 4)] compared to NTG controls [5.84 ± 0.01 (N = 8)] with no differences in Hill coefficients and a small decrease in max tension generation (18.30 ± 1.87 vs. 23.86 ± 1.44) (Figures 1A, D–F and Supplementary Table S1). At P14 TG fibers continued to show increased myofilament Ca^2+^ sensitivity compared to NTG [5.97 ± 0.02 (N = 13) vs. 5.77 ± 0.02 (N = 10)] with decreased Hill coefficient (2.71 ± 0.08 vs. 4.82 ± 0.34) and no difference in max tension (Figures 1B, D–F and Supplementary Table S1). At P28 TG fibers were still more sensitive to Ca^2+^ [6.16 ± 0.05 (N = 5) vs. 5.80 ± 0.02 (N = 5)] and had a decreased Hill coefficient (2.81 ± 0.17 vs. 4.39 ± 0.27) with no changes in max tension when compared to NTG fibers (Figures 1C–F and Supplementary Table S1). Moreover, TG fibers at P28 showed an increased pCa_50_ when compared with TG P14 and TG P7 fibers.

Figure 2A shows a time-dependent expression of TnT variants (TnT3, TnT4 and myc-R92Q) from P2 to P28. Myofilaments from NTG hearts contained low levels of TnT3 and high levels of TnT4. However, TG myofilaments expressed all three variants of TnT. A small amount of myc-TnTR92Q was detected at P2 and reached the maximum level around P10. During this time expression of TnT3 and TnT4 declined. At P10 TG hearts expressed only TnT4 and myc-TnTR92Q. At P7 TG myofilaments expressed 47.6% ± 6.3% (N = 2) of TnT as cTnT-R92Q and this level increased to 90% ± 2.6% (N = 2) by P28.

Expression of α- and β-MHC isoforms was similar in NTG and TG myofilaments from P2 to P28 (Figure 2B). Figure 2C presents changes in the expression of TnI isoforms from P2 to P28. From P2 to P4, the TG and NTG myofilaments expressed only the fetal/neonatal isoform of TnI, slow skeletal troponin I (ssTnI). At P7 both TG and NTG myofilaments expressed 88.9% ± 2.3% and 92.0% ± 1.0% of ssTnI, respectively. At P10 expression of ssTnI was higher in NTG myofilaments compared to TG [32.7% ± 4.8% (N = 4) vs. 11.62% ± 2.89% (N = 4); *p* = 0.0096] indicating a potential influence of cTnT-R92Q expression on thin filament maturation.

### 3.2 Early onset of morphological changes, cardiac and coronary flow dysfunction in TG hearts

**FIGURE 3:**
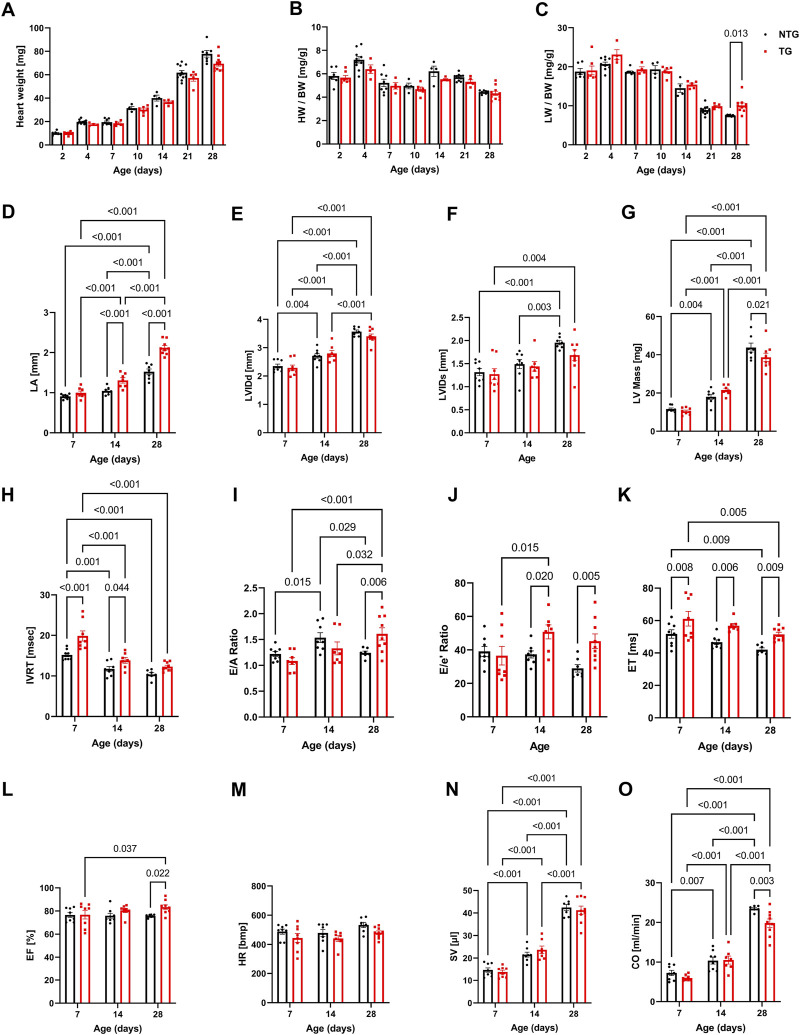
Morphological, systolic and diastolic changes in NTG and HCM hearts. Summary data represent: **(A)** heart weight, **(B)** heart weight to body weight (HW/BW), **(C)** lung weight to body weight (LW/BW), **(D)** left atrial diameter (LA), **(E)** left ventricular internal diastolic diameter (LVIDd), **(F)** left ventricular internal systolic diameter (LVIDs), **(G)** left ventricular mass calculated based on echocardiography (LV Mass), **(H)** isovolumic relaxation time (IVRT), **(I)** E/A ratio represents peak velocity of early diastolic mitral flow divided by peak velocity of late diastolic mitral inflow, **(J)** E/e’ ratio represents peak velocity of early diastolic transmitral flow divided by peak velocity of early diastolic mitral annual motion, **(K)** ejection time (ET), **(L)** ejection fraction (EF), **(M)** heart rate (HR), **(N)** stroke volume (SV), and **(O)** cardiac output. Data reported as mean ± SEM. N = 4–9 for panels **(A–D)**. N = 6–9 for data presented in panels **(E–O)**. Data were analyzed by 2-way ANOVA followed by Fisher’s LSD test; NTG, non-transgenic; TG, transgenic.

To provide a physiologic context to the changing myofilament biophysical interactions, we compared morphological and functional changes in the hearts of NTG and TG mice with complementary changes in coronary flow during these stages of neonatal development. Figures 3A–C and Supplementary Figure S2 summarize cardiac gross morphology and geometry at P2 to P28 in NTG and TG mice. Compared to NTG, TG mice demonstrated similar gross morphologic changes during neonatal growth. However, at P28 we also observed a small increase in lung weight/body weight (LW/BW) in TG compared to NTG mice (Figure 3C). Cardiac geometry and hemodynamic parameters were determined at P7, P14, and P28 in both groups using trans-thoracic echocardiography (Figures 3D–O and Supplementary Table S2). Echocardiography measurements demonstrated an increase in left atrial (LA) size in TG mice compared to NTG at P14 and P28 but not at P7 (Figure 3D). There were no differences in left ventricular internal diameter in diastole (LVIDd) or relative ventricular wall thickness (RWT) between TG and NTG hearts over the 28-day period (Figures 3E, F). However, LV mass determined by echocardiography was significantly smaller at P28 (Figure 3G).

Changes in diastolic function were evaluated using pulsed and tissue Doppler echocardiography (Figures 3H–J and Supplementary Figure S9). A decline in diastolic function of TG hearts was demonstrated by prolonged isovolumic relaxation times (IVRT) as early as at P7 and was also present at P14 (Figure 3H). The E/A ratio, peak velocity of early diastolic mitral inflow divided by peak velocity of late diastolic mitral inflow, was increased in TG compared to NTG hearts at P28 (Figure 3I), and the E/e’ ratio, peak velocity of early mitral inflow over early mitral annular velocity, increased at P14 and P28 in TG hearts (Figure 3J), confirming the development of progressive diastolic dysfunction.

Figures 3K–O summarizes parameters related to cardiac output (CO) in the TG and NTG hearts during neonatal development. Ejection time (ET) was consistently prolonged in TG mice relative to NTG, but ejection fraction (EF) was increased only at P28 (Figures 3K, L) with no significant changes in LVIDd and LVIDs. Although these parameters did not reach significance both parameters were lower in TG hearts. LVIDs were 1.96 ± 0.05 mm in NTG and 1.68 ± 0.12 mm in TG with *p* = 0.06. We observed an age-dependent increase in stroke volume (SV) for the developing hearts, but no differences between TG and NTG hearts at each studied time point. Heart rate (HR) was not significantly different at P7 and P14. However, it was trending towards being lower in TG mice at P28 (*p* = 0.083) (Figure 3M) which resulted in lower CO in TG hearts compared to NTG at P28 (Figure 3O).

**FIGURE 4:**
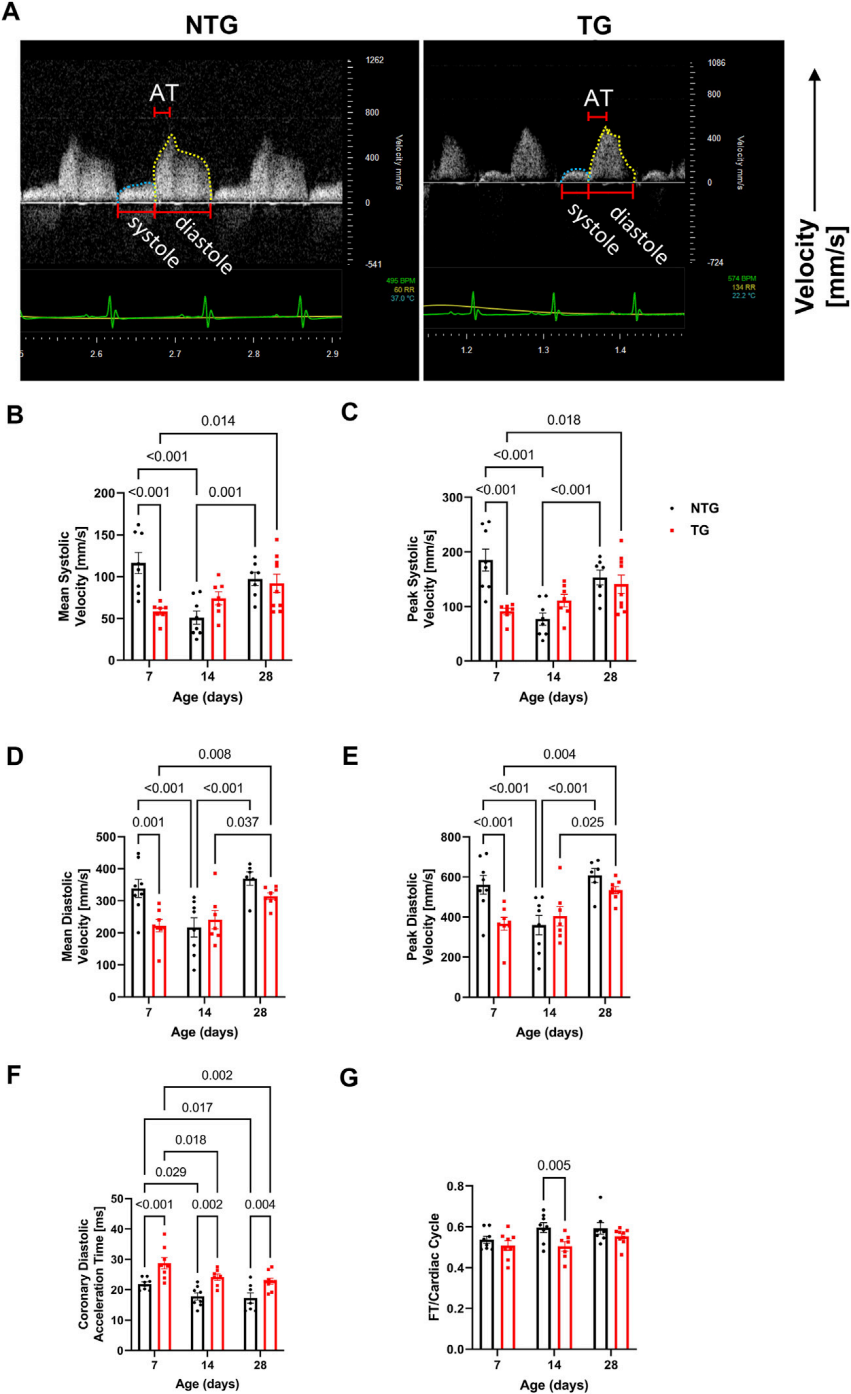
Time-dependent changes in coronary flow. **(A)** Representative Doppler echo images of P14 coronary flow charts, with systolic flow highlighted in blue and diastolic in yellow. Summary data represent: **(B)** mean systolic coronary flow velocity, **(C)** peak systolic coronary flow velocity, **(D)** mean diastolic coronary flow velocity, **(E)** peak diastolic coronary velocity, **(F)** coronary diastolic acceleration time, and **(G)** total diastolic flow time (FT) normalized to cardiac cycle length. Data are presented as mean ± SEM, NTG *n* = 6, TG *n* = 6 and analyzed by 2-way ANOVA followed by Fisher’s LSD test; NTG, non-transgenic; TG transgenic.

Diastolic dysfunction is commonly one of the first alterations seen in HCM patients (Ho et al., 2021; Keam, 2022). However, whether early alterations in relaxation impact coronary flow remains unknown. We, therefore, measured coronary flow dynamics from P7 to P28 in NTG and TG mice (Figure 4 and Supplementary Table S3). As shown in Figure 4A and summarized in Figures 4B–E and Supplementary Table S3, both mean and peak systolic and diastolic flow velocities were significantly decreased in TG hearts relative to NTG at P7 but did not differ at P14 and P28. However, at all ages, TG hearts demonstrated significantly increased diastolic coronary acceleration times (AT) compared to NTG (Figure 4F). Total diastolic flow time (FT) normalized to cardiac cycle length was significantly reduced in TG mice but only at P14 (Figure 4G).

To summarize, expression of TnT-R92Q during the transition of expression from fetal myofilament isoforms to adult isoforms triggers an altered myofilament response to Ca^2+^ that underlies the changes in atrial morphology, diastolic dysfunction, prolonged ejection time, and alterations in coronary flow dynamics. These results also suggest that other signals beyond the defective mechano-transmission of the myofilaments may be contributing to the increased diastolic abnormalities, a stiffer ventricle, and symptomatic heart failure. Since we found early alterations in coronary blood flow, we speculated that decreased vascular perfusion may result in local ischemia and the onset and progression of fibrosis.

### 3.3 Detection of fibrosis

**FIGURE 5:**
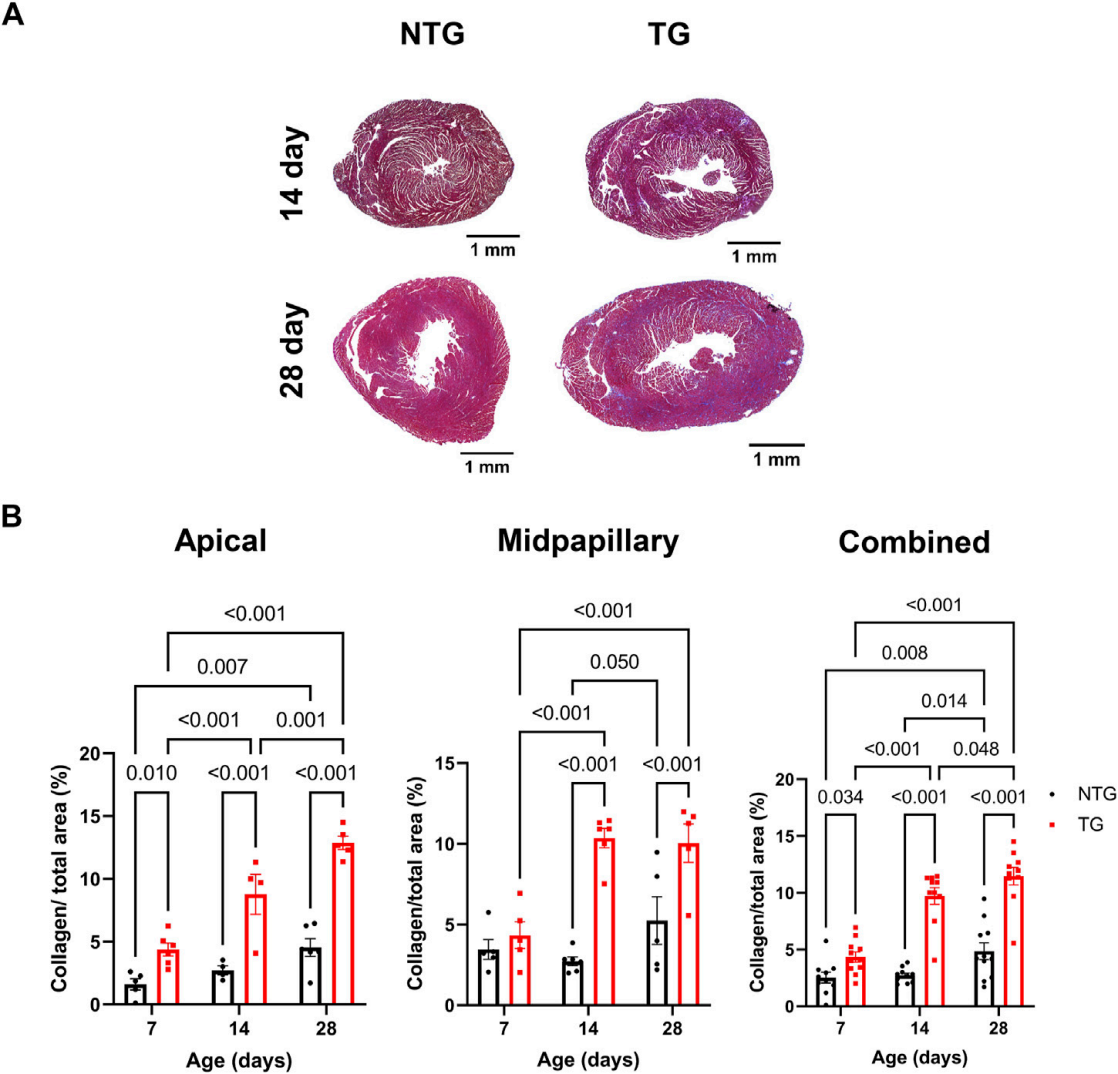
Time-dependent changes in fibrosis in apical and midventricular regions of NTG and TG hearts. **(A)** Representative trichrome-stained midpapillary images of NTG and TG hearts at P14 and P28. **(B)** Quantitation of collagen deposition presented as % of covered area at P7, P14, and P28 at apical, midpapillary and total (apical and midpapillary regions combined). Data are presented as mean ± SEM. Data were analyzed by 2-way ANOVA followed by Fisher’s LSD test; NTG *n* = 4–6, TG *n* = 5–6; NTG, non-transgenic; TG, transgenic.

**FIGURE 6:**
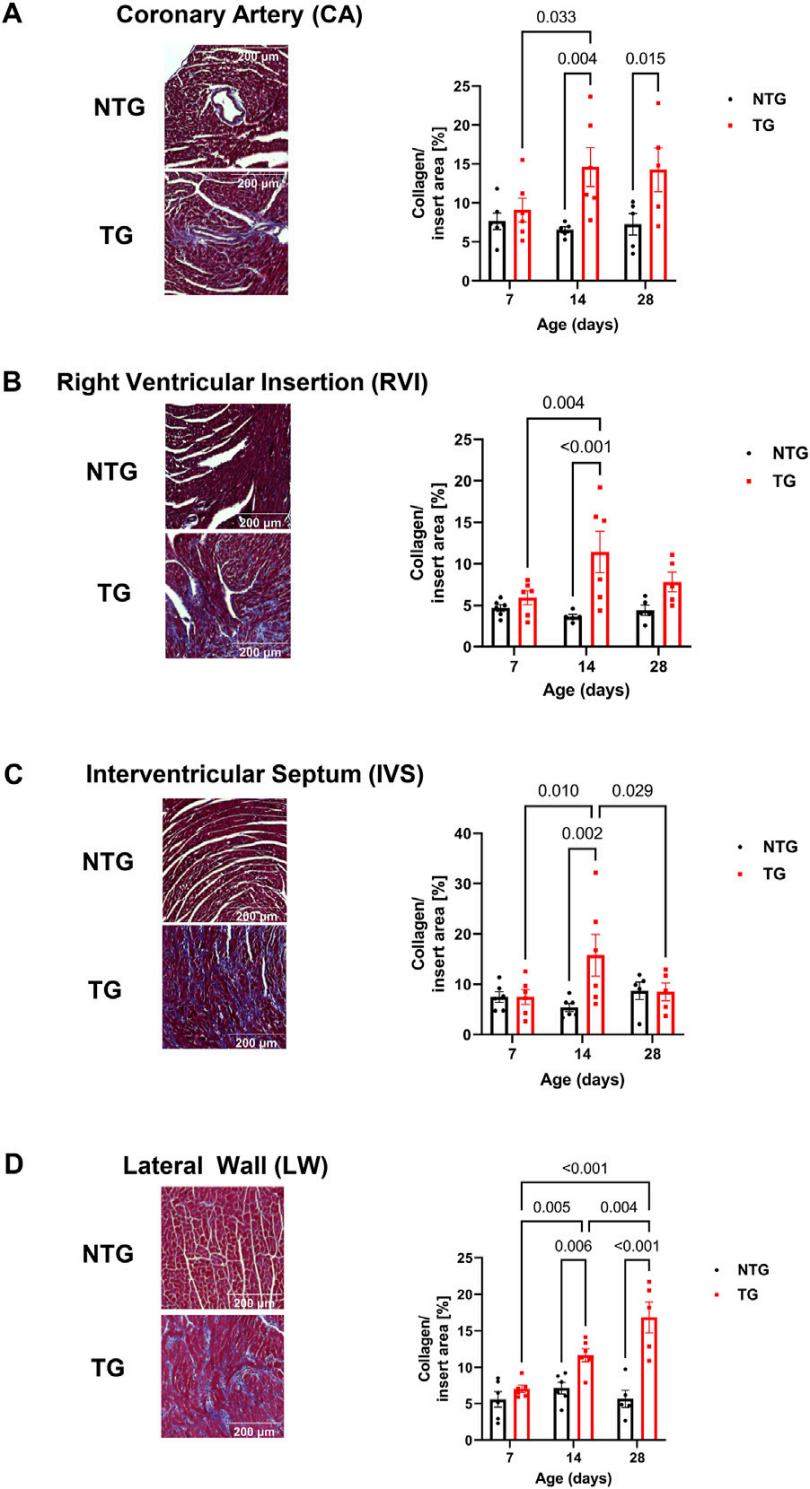
Time-dependent assessment of localized fibrosis. Representative images of areas with high levels of collagen deposition and quantitation of average collagen deposition in four specific locations: **(A)** coronary arteries, **(B)** right ventricular insertion (RVI), **(C)** interventricular septum (IVS) and **(D)** lateral free wall (LW). Data presented as mean ± SEM and analyzed using Two-way ANOVA followed by Fisher’s LSD test; NTG *n* = 5–6, TG *n* = 5–6. NTG, non-transgenic; TG, transgenic.

We have previously reported fibrosis present in four-month-old TG hearts expressing TnT-R92Q (Chowdhury et al., 2020). However, whether fibrosis occurs during neonatal development is unknown. Consequently, we measured cardiac fibrosis by trichrome-staining as a measure of collagen deposition. Representative mid-papillary cross-sections of TG and NTG hearts at P14 and P28 are presented in Figure 5A. Quantifications of total collagen over total cross-sectional area in histological sections from the apical, midpapillary and combined regions of NTG and TG hearts are shown in Figure 5B. We found a significant deposition of collagen that was apparent in the apical and combined sections of TG hearts compared to NTG at all ages. Collagen content was increased at the midpapillary level starting at P14. In focusing on collagen deposition in specific areas, we found evidence at P14 of increased collagen deposition in coronary arteries (CA), right ventricular insertion (RIV), interventricular septum (IVS), and lateral free wall (LW) (Figures 6A–D). These data indicate that the increased fibrosis during early HCM development is localized to specific areas associated with restrictive cardiomyopathy. Moreover, coronary arterial perivascular fibrosis is particularly important as it relates to the coronary flow anomalies we identified.

### 3.4 Micro-vessel density and constrained arteriolar cross-sectional area

**FIGURE 7:**
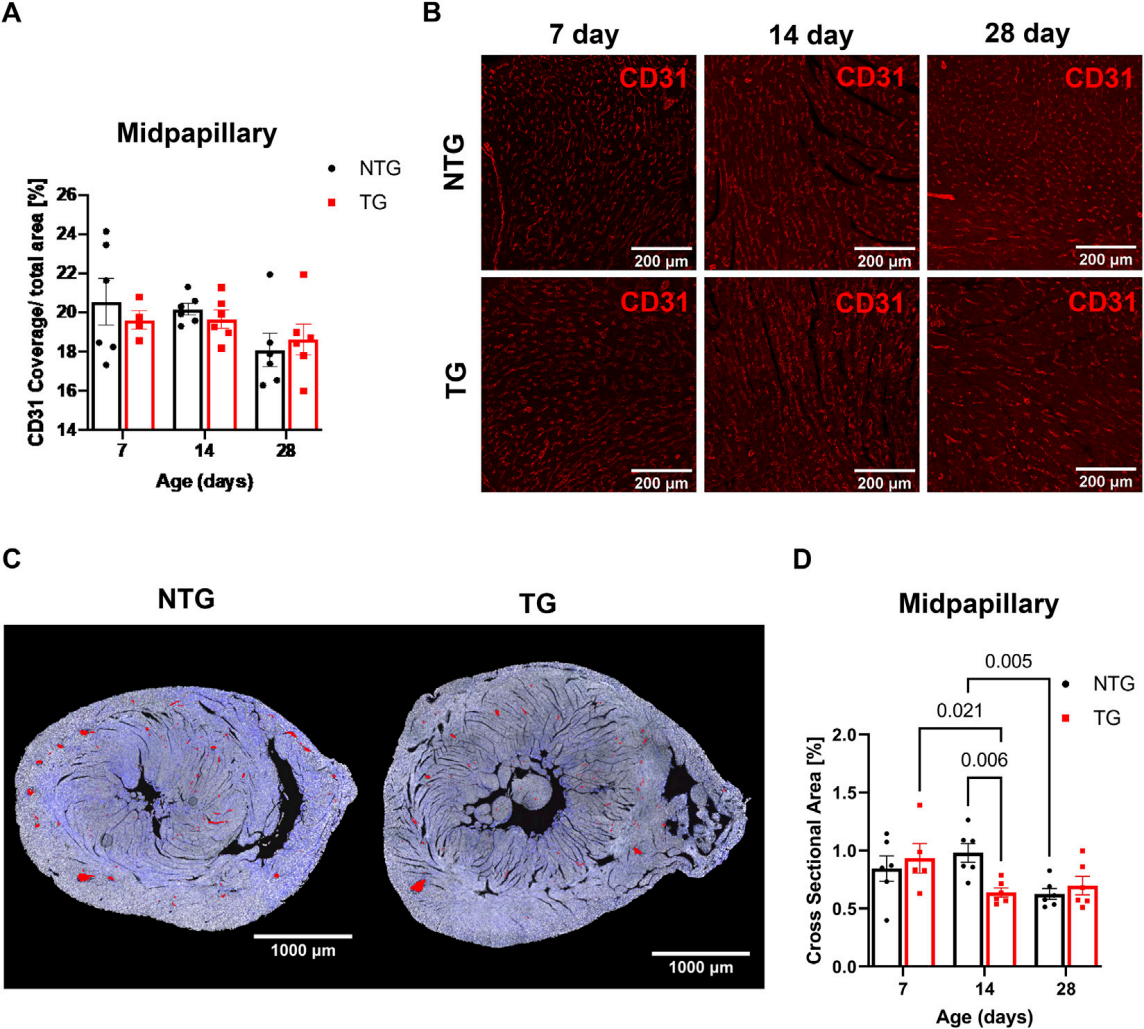
Microvessel density and arteriolar cross-sectional area at midpapillary section. **(A)** Quantitation of CD31 coverage in NTG and TG hearts. **(B)** Representative images photomicrographs of CD31 labeled (indicating endothelial cells) immunofluorescent sections taken at midpapillary levels. **(C)** Representative photomicrographs of midpapillary sections of whole-heart sections with arterioles are highlighted in red at P14. **(D)** Quantification of arteriolar cross-sectional area (%). N = 5–6. Data presented as mean ± SEM; Two-way ANOVA followed by Fisher’s LSD test.

In addition to the impact of perivascular fibrosis on coronary flow, we investigated whether changes in micro-vessel density and arteriolar cross-sectional area could be contributing factors. CD31 staining was used to identify endothelial cells to determine vessel coverage. We found no differences in total vessel density (as represented by CD31 coverage) at any age at the midpapillary level between TG and NTG hearts (Figures 7A, B). However, we did find the total arteriolar cross-sectional area was decreased in TG hearts compared to NTG at P14 within the same midpapillary regions (Figures 7C, D).

### 3.5 YAP signaling in early induction of signaling cascades in hearts expressing cTnT-R92Q

**FIGURE 8:**
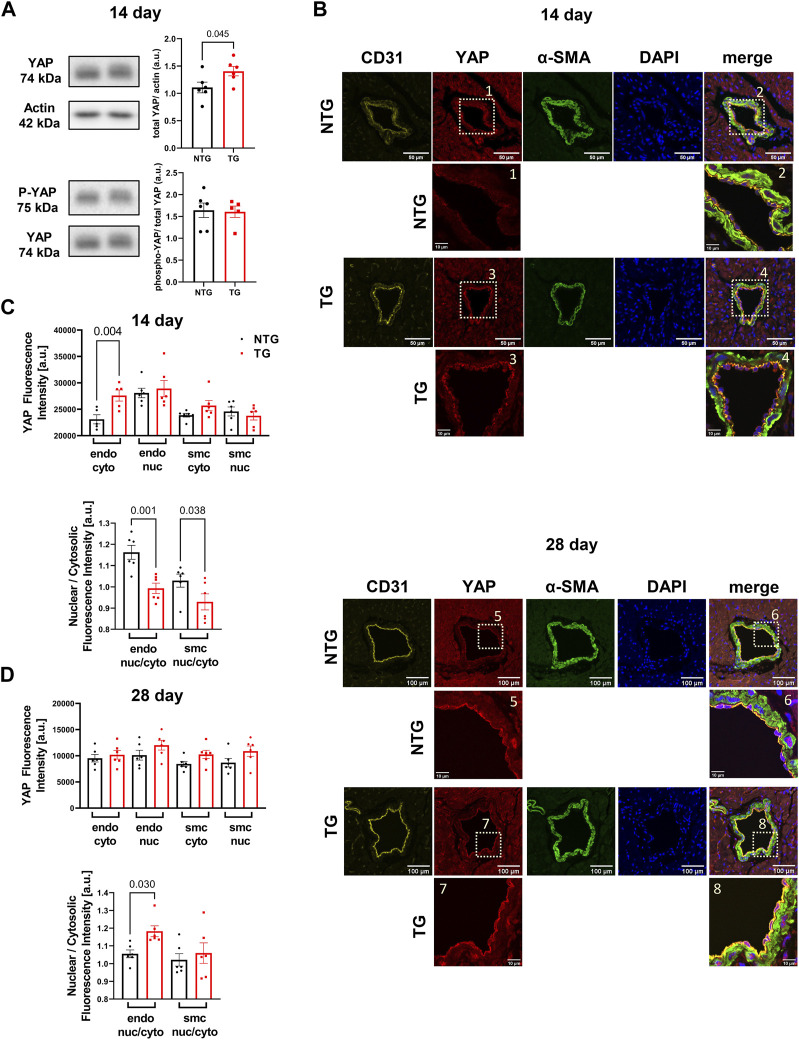
Time-dependent expression and localization of YAP. Western blot analysis of YAP abundance (upper panel) and phosphorylation (p-YAP) (Ser-127) (lower panel) at P14 in TG and NTG hearts **(A)**; Representative immunohistochemistry (IHC) images of coronary vessels from NTG and TG hearts stained with fluorescent antibodies against CD31, YAP, α-SMA and DAPI. The merged images represent CD31/YAP/α-SMA with nuclear DAPI counterstaining of the coronary artery region at the basal section of the heart at P14 **(B)**. Inserts below were taken from YAP and merged images at higher magnification; Cytosolic and nuclear YAP fluorescence signal in endothelial (endo) and smooth muscle cells (smc) in the heart sections and a ratio of nuclear/cytosolic YAP expression in the endothelial and smooth muscle cells (smc) in the heart sections at P14 **(C)**, and P28 **(D)**. Data presented as mean ± SEM and analyzed using Student’s t-test **(A)**, NTG N = 6, TG N = 6 and One-way ANOVA followed by Fisher’s LSD test. NTG N = 5–6, TG N = 5–6 **(C,D)**. NTG, non-transgenic; TG, transgenic.

Involvement of the Hippo pathway with YAP and TAZ as the primary sensors for mechano-transduction signaling has been identified as a potentially significant mechano-sensing mechanism in septal human heart samples in late stage HCM and in pressure/overload hypertrophic growth with up and downregulation of Hippo signaling in cardiac myocytes (Wang et al., 2014; Byun et al., 2019). Moreover, Nakajima et al. (2017) employed zebra fish in studies identifying that blood flow controls the spatiotemporal and transcriptional activation of Yap1 in endothelial cells in live fish. Given the lack of studies of Hippo signaling in early stage HCM together with our findings of alteration in the vascular compartment, we analyzed YAP expression and phosphorylation at site S127 in heart homogenates. Our data showed a transient increase in total YAP expression that occurred at P14 but with no change in phosphorylation (Figure 8A). To identify the spatial abundance of the YAP signal we stained transverse heart sections from the base of P14 TG and NTG hearts and found the increased YAP signal was localized to the endothelium of coronary arteries in the TG mice (Figure 8B, top panel). Analysis of the YAP signal intensity within the endothelial cells showed a significant increase in the cytoplasmic YAP expression in the P14 hearts of TG mice compared to NTG, but with no change in the endothelial nuclear YAP signal (Figure 8C, top panel). Subsequently, we found a decrease in the nuclear/cytosolic YAP expression ratio in the coronary endothelial and smooth muscle cells of P14 TG mice compared to NTG (Figure 8C, bottom panel). Immunohistological staining of P28 hearts showed no differences in the cytosolic and nuclear YAP fluorescence signal in endothelial and smooth muscle cells (Figure 8B, bottom panel; Figure 8D) with no changes in total abundance of YAP and phosphorylation of YAP assessed by immunoblotting (Supplementary Figure S4). However, by P28, the ratio of nuclear/cytosolic YAP expression in the endothelium was increased in TG hearts (Figure 8D). At P7 no changes in YAP localization (Supplementary Figure S3) or abundance (Supplementary Figure S4) were observed. Supplementary Figure S10 shows representative immunohistochemistry images of coronary vessels from NTG and TG hearts stained with fluorescent secondary antibodies (no primary antibody).

### 3.6 Calcineurin, PKA, ERK and Ca^2+^ signaling

**FIGURE 9:**
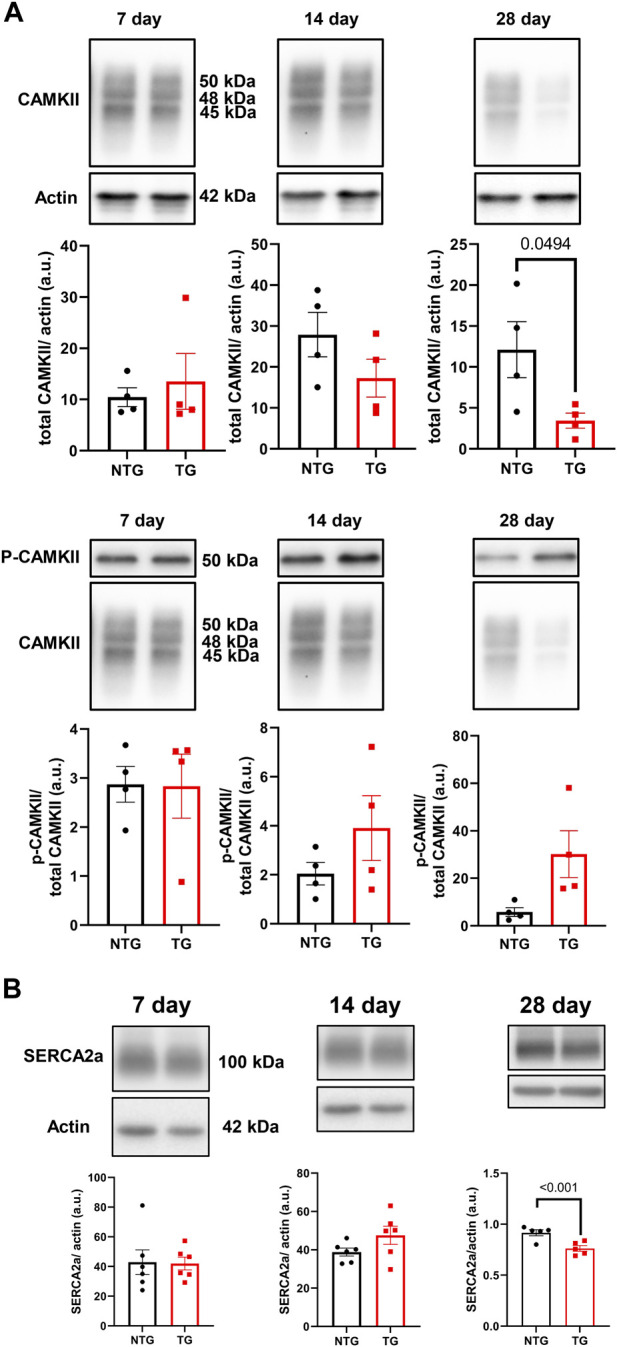
The effects of R92Q-cTnT on Calcium Signaling—CAMKII and SERCA2a abundance. **(A)** Western blot analysis of CAMKII abundance and phosphorylation of CAMKII and **(B)** SERCA2a abundance in NTG and TG hearts. NTG, non-transgenic; TG, transgenic. Data presented as mean ± SEM and analyzed using unpaired Student’s t-test, NTG N = 6, TG N = 5–6.

**FIGURE 10:**
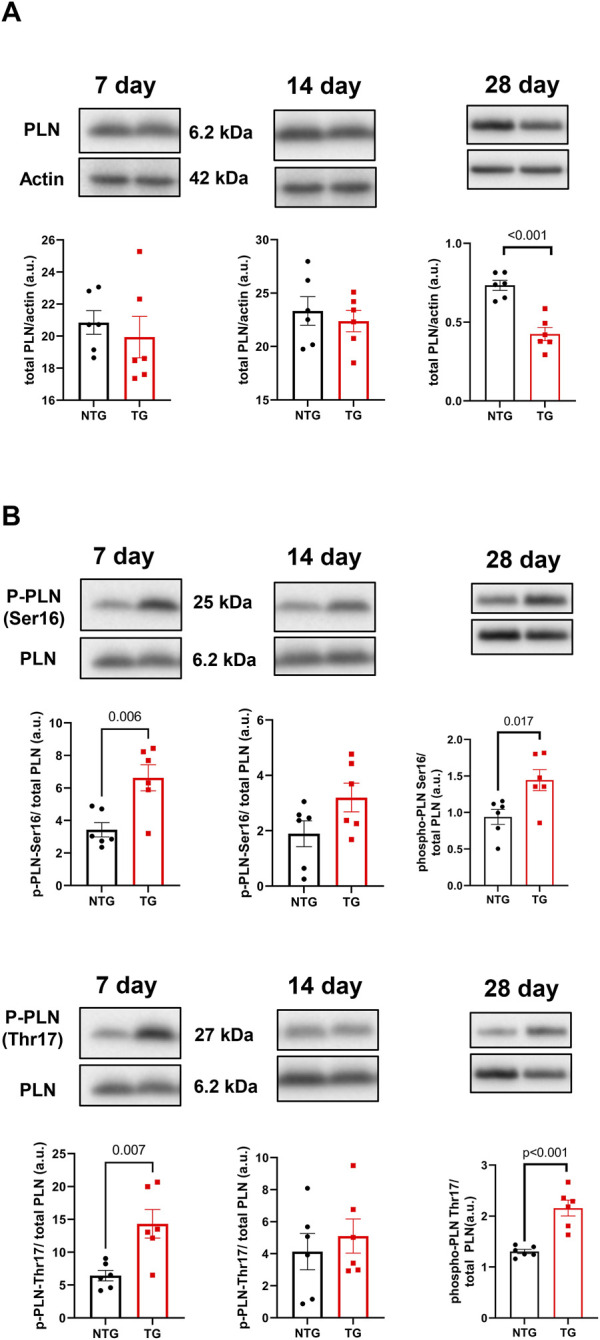
The effects of R92Q-cTnT on calcium signaling—phospholamban (PLN) abundance and phosphorylation. Western blot analysis of PLN abundance **(A)** and phosphorylation of PLN at Ser16 and Thr17 **(B)** in NTG and TG hearts. NTG, non-transgenic; TG, transgenic. Data presented as mean ± SEM and analyzed using unpaired Student’s t-test, NTG N = 6, TG N = 5–6.

Alterations in calcineurin (Supplementary Figure S5A), ERK 1/2 (Supplementary Figures S6A, B), CaMKII (Figure 9A) and Ca^2+^ signaling in late stages of HCM have been previously reported by us and others (Ferrantini et al., 2017; Chowdhury et al., 2020). We have also reported that early corrections in Ca^2+^ signaling can normalize phenotypic changes in TnTR92Q mice (Chowdhury et al., 2020). Consequently, we determined if these signaling proteins were altered early in HCM development. We found a transient decrease in calcineurin expression only at P14, along with no changes in PKA-C and p-PKA-C (Supplementary Figure S5B). Although we did not find changes in ERK1 and ERK2 phosphorylation, the expression of both ERK1 and ERK2 was increased at P14 and P28 (Supplementary Figures S6A, B). Interestingly however, temporal changes in PLN phosphorylation at Ser16 and Thr17 were found at P7 and P28, with the latter associated with a decrease in PLN expression (Figure 10). These changes were accompanied by a trend to increase in the phosphorylation of CaMKII (Figure 9A) and reduced expression of SERCA2a (Figure 9B) by P28.

### 3.7 Oxidative stress

Prior studies with another HCM model expressing mutant tropomyosin (Tm-E180G), reported a role for oxidative stress in HCM phenotype involving NOX2 and NOX4 (Ryba et al., 2019). Moreover, treatment with N-acetylcysteine reversed the diastolic dysfunction associated with diminished S-glutathionylation of MyBP-C (GS-MyBP-C) in this model (Wilder et al., 2015). To examine oxidative stress in the early response to cTnT-R92Q, we compared the activation of endothelial-NOS (eNOS), NOX4, and NOX2 (gp91phox/Nox2) in TG and NTG hearts at different ages (Supplementary Figure S7). We detected only a transient increase of eNOS expression at P7 and NOX2 expression at P14, otherwise no persistent changes were apparent.

### 3.8 Early modifications of myofilaments proteins

**FIGURE 11:**
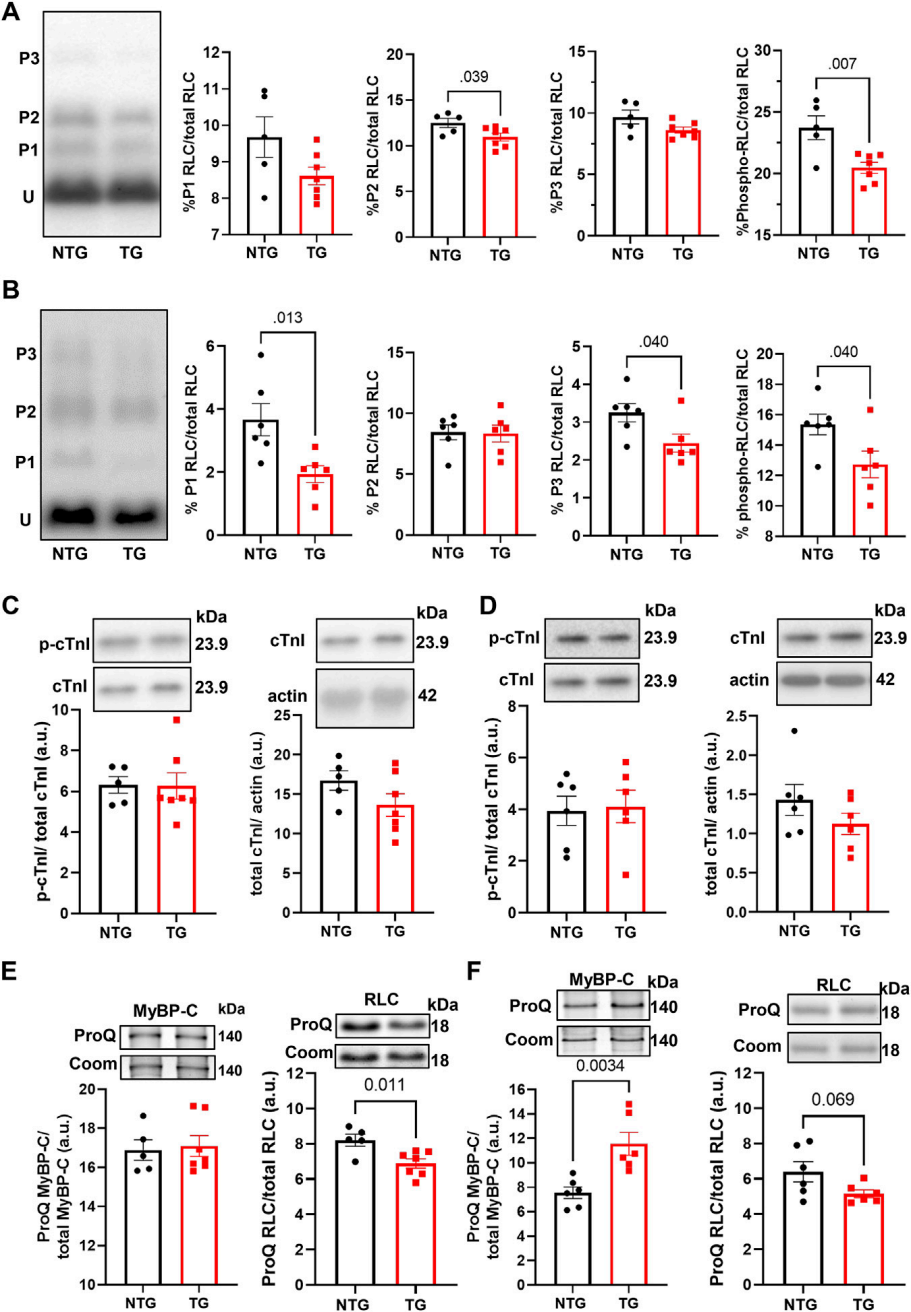
The Effects of R92Q-cTnT and age on myofilament phosphorylation. **(A)** PhosTag separation of RLC at P7. Left most panel representative PhosTag Western blot of RLC phosphorylated species (P1-P3) and unmodified RLC (U). The histograms depict the densitometric analysis of relative percent phosphorylation from the left most representative image. **(B)** PhosTag separation of RLC at P14. Left most panel representative PhosTag Western blot of RLC phosphorylated species (P1-P3) and unmodified RLC (U). The histograms depict the densitometric analysis of relative percent phosphorylation from the left most representative image. **(C)** Western blot analysis of cardiac troponin I (cTnI) and its phosphorylation abundance at positions 23/24 at P7. The left most panel is an analysis of the phosphorylation abundances while the right most panel is the analysis of total troponin I abundance. Images above the histograms are representative Western blot images. **(D)** Western blot analysis of cardiac troponin I (cTnI) and its phosphorylation abundance at positions 23/24 at P14. The left most panel is an analysis of the phosphorylation abundances while the right most panel is the analysis of total troponin I abundance. Images above the histograms are representative Western blot images. **(E)** Heart samples from P7 mice were separated in a 15% SDS-PAGE gel stained with Pro-Q Diamond phospho-specific stain and Coomassie G-250 total protein stain. The left most panel is the analysis of myosin binding protein-C (MyBP-C) phosphorylation abundance and the right most panel is the analysis of regulatory light chain. **(F)** Heart samples from P14 were separated in a 15% SDS-PAGE gel stained with Pro-Q Diamond phospho-specific stain and Coomassie G-250 total protein stain. The left most panel is the analysis of myosin binding protein-C (MyBP-C) phosphorylation abundance and the right most panel is the analysis of regulatory light chain. Data are presented as mean ± SEM and analyzed using unpaired Student’s t-test; N = 5–7.

Finally, we determined the phosphorylation state of myofilament proteins (Figure 11) due to their likely contributions to myofilament Ca^2+^ sensitivity (Figure 1). There was a decrease in total RLC phosphorylation in TG compared to NTG myofibrils at P7 and P14 (Figures 11A, B), and increased phosphorylation of MyBP-C P14 (Figure 11E), which may represent a compensatory response to the increased myofilament Ca^2+^ sensitivity during this developmental phase to the presence of TnT-R92Q. However, no changes in MyBP-C *S*-glutathionylation at P7 and P14 were detected (Supplementary Figure S8).

## 4 Discussion

The important and novel findings described here are that expression of cTnT-R92Q in early postnatal days triggers simultaneous dysfunction in both vascular and cardiac compartments. Our study provides insights into the multiple pathological mechanisms involved in early HCM progression: early coronary flow and diastolic dysfunction, peri-vascular and interstitial fibrosis, compensatory mechanisms at the level of the myofilaments, early induction of adaptive and maladaptive Ca^2+^- and mechano-transduction signaling.

### 4.1 Significance of myofilament protein isoform switching in early-stage HCM

Although changes in myofilament fetal isoforms have been extensively studied during postnatal growth and heart failure (Lowes et al., 1997; Narolska et al., 2005), the impact on the early stages of HCM progression is unknown. While the α-MHC isoform is present in adult mice, the β-MHC isoform is expressed during development and re-expressed with pathology (Narolska et al., 2005). The β-MHC has relatively low myofilament ATP hydrolysis, which slows the velocity of contraction, and improves the efficiency of energy consumption (Rundell et al., 2005; Hoyer et al., 2007). Substituting α-MHC with β-MHC in TG mice expressing cTnT-R92Q rescues the HCM phenotype improves whole heart energetics and decreases the cost of contraction (Rice et al., 2010; He et al., 2012). Our data show that expression of β-MHC decreases to near zero around P10 (Figure 2B) in both NTG and TG hearts but may have some protective effect during the initial development of the HCM phenotype. In addition, we also found that switching from ssTnI to cTnI occurs faster in TG compared to NTG hearts (Figure 2C). Our previous publications reported that the presence of neonatal ssTnI isoform results in an increase in myofilament Ca^2+^ sensitivity, a decrease in sensitivity to acidic pH, ischemia, pressure overload, and stabilization of a glycolytic phenotype (Kobayashi and Solaro, 2005; Solaro et al., 2013). Since ssTnI has multiple effects on cardiac function, this faster switch to cTnI may have either protective or maladaptive effects. At P7 we found an increased myofilament Ca^2+^ sensitivity in cTnT-R92Q compared to controls close to that observed at P14 despite a higher level of mutant protein expression. Based on Phos-Tag gels, at P7 and P14, cTnT-R92Q-myofilaments had decreased MLC2 phosphorylation, but this change did not reach a *p*-value <0.05 at P14 using ProQ gels (Figure 11). In TG-TmE180G hearts, we found significant oxidative stress exacerbating the increase in myofilament Ca^2+^ sensitivity, relieved by sphingosine signaling-induced downregulation of NOX2 (Ryba et al., 2019). In the current study, we found only a transient increase in NOX2 expression at P14, but no changes in S-glutathionylation of MyBP-C (Supplementary Figure S8). Our earlier studies of 16-week-old TG-cTnT-R92Q also showed no changes in MyBP-C S-glutathionylation (Chowdhury et al., 2020). Therefore, the observed increase in myofilament Ca^2+^ sensitivity in TG hearts is a result of TnT-R92Q and neonatal myofilament isoform expression, whereas post translational modifications play a lesser role than previously reported. Consequently, the aberrant biophysical signals triggered by the expression of the mutant protein appear to exert a more direct effect on impaired relaxation. This altered sensitivity and diastolic dysfunction leads to alterations in coronary blood flow.

### 4.2 Diastolic dysfunction and coronary flow

Diastolic dysfunction without left ventricular hypertrophy is an early finding in children with HCM (Poutanen et al., 2006) and animal models mimicking the human phenotype of HCM (Alves et al., 2014). Moreover, a combination of echocardiographic and tissue Doppler image parameters allows the identification of mutant carriers with high sensitivity and specificity (Gandjbakhch et al., 2010). Clinical studies in HCM patients using coronary reserve suggest that the microvascular dysfunction is likely multifactorial (Camici et al., 1991; Krams et al., 1998; Olivotto et al., 2011; Raphael et al., 2016) and includes reduced capillary density, vascular remodeling, fibrosis, myocyte disarray, extravascular compression, diastolic dysfunction and left ventricular outflow tract (LVOT) obstruction (reviewed by Aguiar Rosa et al., 2021).

In our experimental conditions, the diastolic dysfunction observed as early as at P7 establishes a period that represents the time when carriers in humans can be identified. We speculate that one of the observed consequences of this early diastolic dysfunction (Figure 3) is alterations in coronary flow dynamics (Figure 4 and Supplementary Table S3). As a majority of coronary perfusion and flow occurs during diastole, diastolic function is crucial to cardiac sustainability and proper perfusion. Increases in IVRT (Figure 3H) correspond closely to increases in coronary diastolic acceleration time (Figure 4F). Because of this declining diastolic function, mean and peak coronary flow velocities were diminished (Figure 4). Apart from diastolic function and coronary flow, there were no other significant differences at P7 in morphological or functional parameters, emphasizing the critical dependence of coronary flow on diastolic function. Moreover, we did not detect any changes in microvessel density and arteriolar cross-sectional area at this age (Figure 7). However, small decreases in the cross-sectional area were observed at P14 together with an increase in coronary diastolic acceleration time.

### 4.3 Myocardial fibrosis and diastolic dysfunction

HCM patients often develop diastolic dysfunction without severe LVOT obstruction that is associated with diffuse and regional myocardial fibrosis. Using post-contrast analysis of cardiovascular magnetic resonance with late gadolinium enhancement, Ellims et al. (2012) concluded that the diffuse myocardial fibrosis in HCM patients correlates closely with LV filling pressure and diastolic dysfunction. Interestingly, Varnava et al. (2000) further suggested that whereas mutations can induce myocyte disarray directly, the promotion of fibrosis and micro-circulatory disease is a secondary phenomenon unrelated to the disarray. Myocardial fibrosis is commonly found in heart samples obtained at autopsy of young individuals with HCM that succumb to sudden cardiac death. Junttila et al. (2018) reported that a large proportion of subjects with fibrosis had variants in genes associated with different types of cardiomyopathies without myocyte pathology. Other reports characterized regions of diffuse, patchy fibrosis occurring at points of RV insertion (Choudhury et al., 2002). The presence of fibrosis in TnT-R92Q mouse hearts as early as P7 correlates well with these human HCM findings. We also found that fibrosis worsened over the next 3 weeks with a patchy pattern localized primarily to the right ventricular insertion points.

A relevant clinical trial (VANISH; Valsartan for Attenuating Disease Evolution in Early Sarcomeric Hypertrophic Cardiomyopathy) tested the effects of valsartan treatment targeting fibrotic signaling early in the onset of HCM progression (Ho et al., 2021). The investigators concluded that AT1-R blockade delayed disease progression as profiled by improvements in diastolic abnormalities, wall thickness and the biomarker N-terminal pro-B-type natriuretic protein. Data reported here strongly support this approach as we found diastolic dysfunction and coronary flow alterations early in HCM progression alongside signs of fibrosis. Our data agree with the outcome of the VANISH trial and further support the value of early-stage therapies targeting fibrosis.

### 4.4 Progression of modifications in YAP signaling

Mechano-sensing by the Hippo pathway within the endothelium provides a novel direction to understanding HCM progression. Our results indicate inside/out and outside/in mechanical stresses are acting on the micro-environment surrounding the vessels and myocytes. Inside/out stresses arise from myocyte hypercontractility, and prolonged compression during systole, whereas outside/in stresses arise from ECM stiffness as well as diastolic dysfunction giving rise to compression, coronary flow abnormalities, and sheer stress. Key terminal effectors in the Hippo pathway are co-transcriptional activators, YAP/TAZ, which shuttle between the cytosol and nucleus (Dupont et al., 2011). Ablation of YAP in cardiac myocytes induces a worsening of pressure overload hypertrophy, hypoxia/oxidative stress, and ischemia/reperfusion injury (Matsuda et al., 2016; Byun et al., 2019). Overexpression of cardiac YAP-S127A in myocytes redistributes the protein more toward the nucleus, which provides cardiac protection in MI (Lin et al., 2014). Within endothelial cells (EC), the HIPPO pathway transduces mechanical signals and modulates proliferation and angiogenesis (Dupont et al., 2011). Accordingly, EC specific deletion of YAP/TAZ results in apoptosis and vascular defects during embryonic development. A deletion during neonatal development results in reduced vascular area, vessel branching, radial outgrowth, and EC proliferation in various tissues, leading to smaller tissue size, including the heart (Wang et al., 2017). Our data showing increased total YAP expression at P14 and associated decrease in the ratio of nuclear to cytosolic YAP indicate that EC mechano-sensing pathways are responding to changes in the micro-environment in TnT-R92Q neonatal hearts. Conversely at P28, the ratio reverted towards a decrease in nuclear to cytosolic YAP signal in endothelial cells. Related to our discovery of increased YAP in the EC cytosol of P14 TnT-R92Q mice are data presented by Sakabe et al. (2017). The authors report that cytoplasmic YAP/TAZ promotes angiogenesis through CDC42 activation, affecting cytoskeletal regulatory mechanisms (Sakabe et al., 2017).

### 4.5 Progression of maladaptive Ca^2+^-signaling

Changes in Ca^2+^ signaling during the early phases of HCM development are understudied. We have previously reported an interplay between cellular Ca^2+^ management and HCM progression in the Tm-E180G mouse model (Pena et al., 2010; Gaffin et al., 2011). We reported protective effects of adenoviral-mediated overexpression of SERCA2a in neonatal mouse hearts and KO of PLN in Tm-E180G. There is substantial evidence connecting increased Ca^2+^ sensitivity in HCM-linked TNNT2 mutations with an effect on Ca^2+^ cycling, Ca^2+^ buffering and arrhythmogenesis (Schober et al., 2012; Coppini et al., 2017; Ferrantini et al., 2017; Smith and Eisner, 2019). Recently, we presented that PLNKO was also beneficial for preventing HCM phenotype in TnT-R92Q mice (Chowdhury et al., 2020). Our current data show that at P7 the phosphorylation levels of PLN at Ser16 and Thr17 were increased suggesting increased SERCA2 activity that can be seen as a compensatory and protective mechanism.

The role of correcting Ca^2+^ signaling in TnT-R92Q mice to prevent the development of the HCM phenotype was reported by Coppini et al. (2017). Early administration of ranolazine, the inhibitor of the late Na^+^ current, resulted in a sustained reduction of intracellular Ca^2+^ and CaMKII activity and prevented the morphological and functional HCM phenotype in TG-cTnT-R92Q mice. The authors attributed ranolazine inhibition of the late Na^+^ current as the likely causative factor, but our earlier studies suggested modifications in myofilament Ca^2+^ response induced by ranolazine as a contender (Lovelock et al., 2012). Moreover, Sharp et al. (2021) summarized evidence that ranolazine, in clinical use for angina, is effective in the treatment of coronary microvascular dysfunction.

The early-stage alterations in cellular Ca^2+^ homeostasis and myofilament Ca^2+^ sensitivity may affect intracellular Ca^2+^ buffering. Since only about 1.0% of Ca^2+^ released from the sarcoplasmic reticulum (SR) is free, Ca^2+^ distributes among buffers that consist mainly of SERCA2a, cTnC and CaM (Smith and Eisner, 2019). In our previous studies, we reported that PLN phosphorylation increases buffering, whereas phosphorylation of TnI decreases Ca^2+^ buffering, and therefore β-adrenergic stimulation of ventricular myocytes did not affect Ca^2+^ buffering as SERCA2a and cTnC were modified to rebalance Ca^2+^ perturbations induced by PLN and cTnI phosphorylation (Briston et al., 2014). In our current studies, we speculate that at P7 phosphorylation of PLN reduces Ca^2+^ buffering by SERCA2a and fosters an environment dominated by cTnC and CaM buffering, as suggested by increases in Thr17 PLN phosphorylation indicating increased Ca^2+^ binding to CaM (Kranias and Hajjar, 2012). Increased myofilament Ca^2+^ sensitivity observed as soon as TnT-R92Q is expressed also further increases Ca^2+^ buffering. Schober et al. (2012) have reported that the increased Ca^2+^ buffering by cTnC in late-stage HCM induces a delay in Ca^2+^ reuptake likely underlying diastolic abnormalities. In addition, the prolonged decay results in elevated diastolic Ca^2+^ with faster heart rates, and post-rest potentiation with induction of pause-dependent arrhythmias (Schober et al., 2012). Similar findings have been reported regarding the relationship between maladaptive Ca^2+^ homeostasis in the late-stage effects on morphology and function of expression cTnT-R92Q in mouse models (Coppini et al., 2017; Ferrantini et al., 2017). Thus, our findings here at 28-day of age in the TG-cTnT-R92Q hearts compared to controls of no net changes in PLN phosphorylation, a decrease in SERCA2a expression, and an increase CaM kinase phosphorylation fit with our findings at a much later stage (Chowdhury et al., 2020).

## 5 Conclusion

Our studies reveal that coronary flow alterations and fibrotic processes in the early stage of TnT-R92Q HCM accompany the diastolic dysfunction induced by increased myofilament Ca^2+^ sensitivity. Our data also stress the importance of crosstalk between cardiac myocytes and other compartments of the heart in TnT-R92Q HCM progression and highlight the need for a deeper understanding and integration of vascular dysfunction in the pathogenesis of HCM. Although we studied one model of HCM, we speculate that early coronary flow alterations will most likely be present if the expression of a mutated myofilament protein is accompanied by early diastolic dysfunction. However, further studies in multiple models of HCM are needed to better understand if these early tissue-level functional and phenotypic are common to all forms of HCM.

## Data Availability

The original contributions presented in the study are included in the article/Supplementary Material, further inquiries can be directed to the corresponding author.

## References

[B1] Aguiar Rosa S. Rocha Lopes L. Fiarresga A. Ferreira R. C. Mota Carmo M. (2021). Coronary microvascular dysfunction in hypertrophic cardiomyopathy: Pathophysiology, assessment, and clinical impact. Microcirculation 28, e12656. 10.1111/micc.12656 32896949

[B2] Alves M. L. Dias F. a. L. Gaffin R. D. Simon J. N. Montminy E. M. Biesiadecki B. J. (2014). Desensitization of myofilaments to Ca2+ as a therapeutic target for hypertrophic cardiomyopathy with mutations in thin filament proteins. Circ. Cardiovasc Genet. 7, 132–143. 10.1161/CIRCGENETICS.113.000324 24585742 PMC4061696

[B3] Anderson P. A. Malouf N. N. Oakeley A. E. Pagani E. D. Allen P. D. (1991). Troponin T isoform expression in humans. A comparison among normal and failing adult heart, fetal heart, and adult and fetal skeletal muscle. Circ. Res. 69, 1226–1233. 10.1161/01.res.69.5.1226 1934353

[B4] Araujo A. Q. Arteaga E. Ianni B. M. Buck P. C. Rabello R. Mady C. (2005). Effect of Losartan on left ventricular diastolic function in patients with nonobstructive hypertrophic cardiomyopathy. Am. J. Cardiol. 96, 1563–1567. 10.1016/j.amjcard.2005.07.065 16310441

[B5] Briston S. J. Dibb K. M. Solaro R. J. Eisner D. A. Trafford A. W. (2014). Balanced changes in Ca buffering by SERCA and troponin contribute to Ca handling during beta-adrenergic stimulation in cardiac myocytes. Cardiovasc Res. 104, 347–354. 10.1093/cvr/cvu201 25183792 PMC4240166

[B6] Byun J. Del Re D. P. Zhai P. Ikeda S. Shirakabe A. Mizushima W. (2019). Yes-associated protein (YAP) mediates adaptive cardiac hypertrophy in response to pressure overload. J. Biol. Chem. 294, 3603–3617. 10.1074/jbc.RA118.006123 30635403 PMC6416448

[B7] Camici P. Chiriatti G. Lorenzoni R. Bellina R. C. Gistri R. Italiani G. (1991). Coronary vasodilation is impaired in both hypertrophied and nonhypertrophied myocardium of patients with hypertrophic cardiomyopathy: A study with nitrogen-13 ammonia and positron emission tomography. J. Am. Coll. Cardiol. 17, 879–886. 10.1016/0735-1097(91)90869-b 1999624

[B8] Cannon L. Yu Z. Y. Marciniec T. Waardenberg A. J. Iismaa S. E. Nikolova-Krstevski V. (2015). Irreversible triggers for hypertrophic cardiomyopathy are established in the early postnatal period. J. Am. Coll. Cardiol. 65, 560–569. 10.1016/j.jacc.2014.10.069 25677315

[B9] Chang W. T. Fisch S. Chen M. Qiu Y. Cheng S. Liao R. (2015). Ultrasound based assessment of coronary artery flow and coronary flow reserve using the pressure overload model in mice. J. Vis. Exp., e52598. 10.3791/52598 25938185 PMC4541549

[B10] Choudhury L. Mahrholdt H. Wagner A. Choi K. M. Elliott M. D. Klocke F. J. (2002). Myocardial scarring in asymptomatic or mildly symptomatic patients with hypertrophic cardiomyopathy. J. Am. Coll. Cardiol. 40, 2156–2164. 10.1016/s0735-1097(02)02602-5 12505229

[B11] Chowdhury S. a. K. Warren C. M. Simon J. N. Ryba D. M. Batra A. Varga P. (2020). Modifications of sarcoplasmic reticulum function prevent progression of sarcomere-linked hypertrophic cardiomyopathy despite a persistent increase in myofilament calcium response. Front. Physiol. 11, 107. 10.3389/fphys.2020.00107 32210830 PMC7075858

[B12] Coppini R. Mazzoni L. Ferrantini C. Gentile F. Pioner J. M. Laurino A. (2017). Ranolazine prevents phenotype development in a mouse model of hypertrophic cardiomyopathy. Circ. Heart Fail 10, e003565. 10.1161/CIRCHEARTFAILURE.116.003565 28255011 PMC6284403

[B13] Dupont S. Morsut L. Aragona M. Enzo E. Giulitti S. Cordenonsi M. (2011). Role of YAP/TAZ in mechanotransduction. Nature 474, 179–183. 10.1038/nature10137 21654799

[B14] Ellims A. H. Iles L. M. Ling L. H. Hare J. L. Kaye D. M. Taylor A. J. (2012). Diffuse myocardial fibrosis in hypertrophic cardiomyopathy can be identified by cardiovascular magnetic resonance, and is associated with left ventricular diastolic dysfunction. J. Cardiovasc Magn. Reson 14, 76. 10.1186/1532-429X-14-76 23107451 PMC3502601

[B15] Ferrantini C. Coppini R. Pioner J. M. Gentile F. Tosi B. Mazzoni L. (2017). Pathogenesis of hypertrophic cardiomyopathy is mutation rather than disease specific: A comparison of the cardiac troponin T E163R and R92Q mouse models. J. Am. Heart Assoc. 6, e005407. 10.1161/JAHA.116.005407 28735292 PMC5586279

[B16] Flenner F. Geertz B. Reischmann-Dusener S. Weinberger F. Eschenhagen T. Carrier L. (2017). Diltiazem prevents stress-induced contractile deficits in cardiomyocytes, but does not reverse the cardiomyopathy phenotype in Mybpc3-knock-in mice. J. Physiol. 595, 3987–3999. 10.1113/JP273769 28090637 PMC5471503

[B17] Foa A. Agostini V. Rapezzi C. Olivotto I. Corti B. Potena L. (2019). Histopathological comparison of intramural coronary artery remodeling and myocardial fibrosis in obstructive versus end-stage hypertrophic cardiomyopathy. Int. J. Cardiol. 291, 77–82. 10.1016/j.ijcard.2019.03.060 30979607

[B18] Gaffin R. D. Pena J. R. Alves M. S. Dias F. A. Chowdhury S. A. Heinrich L. S. (2011). Long-term rescue of a familial hypertrophic cardiomyopathy caused by a mutation in the thin filament protein, tropomyosin, via modulation of a calcium cycling protein. J. Mol. Cell Cardiol. 51, 812–820. 10.1016/j.yjmcc.2011.07.026 21840315 PMC3221410

[B19] Gandjbakhch E. Gackowski A. Tezenas Du Montcel S. Isnard R. Hamroun A. Richard P. (2010). Early identification of mutation carriers in familial hypertrophic cardiomyopathy by combined echocardiography and tissue Doppler imaging. Eur. Heart J. 31, 1599–1607. 10.1093/eurheartj/ehq101 20439259

[B20] He H. Hoyer K. Tao H. Rice R. Jimenez J. Tardiff J. C. (2012). Myosin-driven rescue of contractile reserve and energetics in mouse hearts bearing familial hypertrophic cardiomyopathy-associated mutant troponin T is mutation-specific. J. Physiol. 590, 5371–5388. 10.1113/jphysiol.2012.234252 22907055 PMC3515825

[B21] Ho C. Y. Day S. M. Axelsson A. Russell M. W. Zahka K. Lever H. M. (2021). Valsartan in early-stage hypertrophic cardiomyopathy: A randomized phase 2 trial. Nat. Med. 27, 1818–1824. 10.1038/s41591-021-01505-4 34556856 PMC8666141

[B22] Hoyer K. Krenz M. Robbins J. Ingwall J. S. (2007). Shifts in the myosin heavy chain isozymes in the mouse heart result in increased energy efficiency. J. Mol. Cell Cardiol. 42, 214–221. 10.1016/j.yjmcc.2006.08.116 17054980 PMC4412927

[B23] Junttila M. J. Holmstrom L. Pylkas K. Mantere T. Kaikkonen K. Porvari K. (2018). Primary myocardial fibrosis as an alternative phenotype pathway of inherited cardiac structural disorders. Circulation 137, 2716–2726. 10.1161/CIRCULATIONAHA.117.032175 29915098

[B24] Karbassi E. Fenix A. Marchiano S. Muraoka N. Nakamura K. Yang X. (2020). Cardiomyocyte maturation: Advances in knowledge and implications for regenerative medicine. Nat. Rev. Cardiol. 17, 341–359. 10.1038/s41569-019-0331-x 32015528 PMC7239749

[B25] Keam S. J. (2022). Mavacamten: First approval. Drugs 82, 1127–1135. 10.1007/s40265-022-01739-7 35802255 PMC9338109

[B26] Kobayashi T. Solaro R. J. (2005). Calcium, thin filaments, and the integrative biology of cardiac contractility. Annu. Rev. Physiol. 67, 39–67. 10.1146/annurev.physiol.67.040403.114025 15709952

[B27] Krams R. Kofflard M. J. M. Duncker D. J. Von Birgelen C. Carlier S. Kliffen M. (1998). Decreased coronary flow reserve in hypertrophic cardiomyopathy is related to remodeling of the coronary microcirculation. Circulation 97, 230–233. 10.1161/01.cir.97.3.230 9462521

[B28] Kranias E. G. Hajjar R. J. (2012). Modulation of cardiac contractility by the phospholamban/SERCA2a regulatome. Circ. Res. 110, 1646–1660. 10.1161/CIRCRESAHA.111.259754 22679139 PMC3392125

[B29] Li Y. Charles P. Y. Nan C. Pinto J. R. Wang Y. Liang J. (2010). Correcting diastolic dysfunction by Ca2+ desensitizing troponin in a transgenic mouse model of restrictive cardiomyopathy. J. Mol. Cell Cardiol. 49, 402–411. 10.1016/j.yjmcc.2010.04.017 20580639 PMC5394742

[B30] Lin Z. Von Gise A. Zhou P. Gu F. Ma Q. Jiang J. (2014). Cardiac-specific YAP activation improves cardiac function and survival in an experimental murine MI model. Circ. Res. 115, 354–363. 10.1161/CIRCRESAHA.115.303632 24833660 PMC4104149

[B31] Lovelock J. D. Monasky M. M. Jeong E. M. Lardin H. A. Liu H. Patel B. G. (2012). Ranolazine improves cardiac diastolic dysfunction through modulation of myofilament calcium sensitivity. Circ. Res. 110, 841–850. 10.1161/CIRCRESAHA.111.258251 22343711 PMC3314887

[B32] Lowes B. D. Minobe W. Abraham W. T. Rizeq M. N. Bohlmeyer T. J. Quaife R. A. (1997). Changes in gene expression in the intact human heart. Downregulation of alpha-myosin heavy chain in hypertrophied, failing ventricular myocardium. J. Clin. Invest. 100, 2315–2324. 10.1172/JCI119770 9410910 PMC508428

[B33] Maron B. J. Maron M. S. Maron B. A. Loscalzo J. (2019). Moving beyond the sarcomere to explain heterogeneity in hypertrophic cardiomyopathy: JACC review topic of the week. J. Am. Coll. Cardiol. 73, 1978–1986. 10.1016/j.jacc.2019.01.061 31000001 PMC6550351

[B34] Maron B. J. (2018). Clinical course and management of hypertrophic cardiomyopathy. N. Engl. J. Med. 379, 655–668. 10.1056/NEJMra1710575 30110588

[B35] Matsuda T. Zhai P. Sciarretta S. Zhang Y. Jeong J. I. Ikeda S. (2016). NF2 activates Hippo signaling and promotes ischemia/reperfusion injury in the heart. Circ. Res. 119, 596–606. 10.1161/CIRCRESAHA.116.308586 27402866 PMC4992450

[B36] Mearini G. Stimpel D. Geertz B. Weinberger F. Kramer E. Schlossarek S. (2014). Mybpc3 gene therapy for neonatal cardiomyopathy enables long-term disease prevention in mice. Nat. Commun. 5, 5515. 10.1038/ncomms6515 25463264

[B37] Nagueh S. F. Bachinski L. L. Meyer D. Hill R. Zoghbi W. A. Tam J. W. (2001). Tissue Doppler imaging consistently detects myocardial abnormalities in patients with hypertrophic cardiomyopathy and provides a novel means for an early diagnosis before and independently of hypertrophy. Circulation 104, 128–130. 10.1161/01.cir.104.2.128 11447072 PMC2900859

[B38] Nakajima H. Yamamoto K. Agarwala S. Terai K. Fukui H. Fukuhara S. (2017). Flow-dependent endothelial YAP regulation contributes to vessel maintenance. Dev. Cell 40, 523–536 e6. 10.1016/j.devcel.2017.02.019 28350986

[B39] Narolska N. A. Eiras S. Van Loon R. B. Boontje N. M. Zaremba R. Spiegelen Berg S. R. (2005). Myosin heavy chain composition and the economy of contraction in healthy and diseased human myocardium. J. Muscle Res. Cell Motil. 26, 39–48. 10.1007/s10974-005-9005-x 16088376

[B40] Olivotto I. Girolami F. Sciagra R. Ackerman M. J. Sotgia B. Bos J. M. (2011). Microvascular function is selectively impaired in patients with hypertrophic cardiomyopathy and sarcomere myofilament gene mutations. J. Am. Coll. Cardiol. 58, 839–848. 10.1016/j.jacc.2011.05.018 21835320

[B41] Pena J. R. Szkudlarek A. C. Warren C. M. Heinrich L. S. Gaffin R. D. Jagatheesan G. (2010). Neonatal gene transfer of Serca2a delays onset of hypertrophic remodeling and improves function in familial hypertrophic cardiomyopathy. J. Mol. Cell Cardiol. 49, 993–1002. 10.1016/j.yjmcc.2010.09.010 20854827 PMC2982190

[B42] Poutanen T. Tikanoja T. Jaaskelainen P. Jokinen E. Silvast A. Laakso M. (2006). Diastolic dysfunction without left ventricular hypertrophy is an early finding in children with hypertrophic cardiomyopathy-causing mutations in the beta-myosin heavy chain, alpha-tropomyosin, and myosin-binding protein C genes. Am. Heart J. 151, 725 e1–e725725.e9. 10.1016/j.ahj.2005.12.005 16504640

[B43] Raphael C. E. Cooper R. Parker K. H. Collinson J. Vassiliou V. Pennell D. J. (2016). Mechanisms of myocardial ischemia in hypertrophic cardiomyopathy: Insights from wave intensity analysis and magnetic resonance. J. Am. Coll. Cardiol. 68, 1651–1660. 10.1016/j.jacc.2016.07.751 27712778 PMC5054113

[B44] Rice R. Guinto P. Dowell-Martino C. He H. Hoyer K. Krenz M. (2010). Cardiac myosin heavy chain isoform exchange alters the phenotype of cTnT-related cardiomyopathies in mouse hearts. J. Mol. Cell Cardiol. 48, 979–988. 10.1016/j.yjmcc.2009.11.018 20004663 PMC3016872

[B45] Rundell V. L. Manaves V. Martin A. F. De Tombe P. P. (2005). Impact of beta-myosin heavy chain isoform expression on cross-bridge cycling kinetics. Am. J. Physiol. Heart Circ. Physiol. 288, H896–H903. 10.1152/ajpheart.00407.2004 15471982

[B46] Ryba D. M. Warren C. M. Karam C. N. Davis R. T. 3rd Chowdhury S. a. K. Alvarez M. G. (2019). Sphingosine-1-Phosphate receptor modulator, FTY720, improves diastolic dysfunction and partially reverses atrial remodeling in a Tm-E180G mouse model linked to hypertrophic cardiomyopathy. Circ. Heart Fail 12, e005835. 10.1161/CIRCHEARTFAILURE.118.005835 31684756 PMC6857720

[B47] Sakabe M. Fan J. Odaka Y. Liu N. Hassan A. Duan X. (2017). YAP/TAZ-CDC42 signaling regulates vascular tip cell migration. Proc. Natl. Acad. Sci. U. S. A. 114, 10918–10923. 10.1073/pnas.1704030114 28973878 PMC5642684

[B48] Schober T. Huke S. Venkataraman R. Gryshchenko O. Kryshtal D. Hwang H. S. (2012). Myofilament Ca sensitization increases cytosolic Ca binding affinity, alters intracellular Ca homeostasis, and causes pause-dependent Ca-triggered arrhythmia. Circ. Res. 111, 170–179. 10.1161/CIRCRESAHA.112.270041 22647877 PMC3393041

[B49] Semsarian C. Ingles J. Maron M. S. Maron B. J. (2015). Reply: What is the true prevalence of hypertrophic cardiomyopathy? J. Am. Coll. Cardiol. 66, 1846–1847. 10.1016/j.jacc.2015.07.073 26483114

[B50] Sharp R. P. Patatanian E. Sirajuddin R. (2021). Use of ranolazine for the treatment of coronary microvascular dysfunction. Am. J. Cardiovasc Drugs 21, 513–521. 10.1007/s40256-020-00462-6 33438139

[B51] Smith G. L. Eisner D. A. (2019). Calcium buffering in the heart in health and disease. Circulation 139, 2358–2371. 10.1161/CIRCULATIONAHA.118.039329 31082292 PMC6520234

[B52] Solaro R. J. Henze M. Kobayashi T. (2013). Integration of troponin I phosphorylation with cardiac regulatory networks. Circ. Res. 112, 355–366. 10.1161/CIRCRESAHA.112.268672 23329791 PMC3567448

[B53] Varnava A. M. Elliott P. M. Sharma S. Mckenna W. J. Davies M. J. (2000). Hypertrophic cardiomyopathy: The interrelation of disarray, fibrosis, and small vessel disease. Heart 84, 476–482. 10.1136/heart.84.5.476 11040002 PMC1729476

[B54] Wang P. Mao B. Luo W. Wei B. Jiang W. Liu D. (2014). The alteration of Hippo/YAP signaling in the development of hypertrophic cardiomyopathy. Basic Res. Cardiol. 109, 435. 10.1007/s00395-014-0435-8 25168380

[B55] Wang X. Freire Valls A. Schermann G. Shen Y. Moya I. M. Castro L. (2017). YAP/TAZ orchestrate VEGF signaling during developmental angiogenesis. Dev. Cell 42, 462–478 e7. 10.1016/j.devcel.2017.08.002 28867486

[B56] Warren C. M. Greaser M. L. (2003). Method for cardiac myosin heavy chain separation by sodium dodecyl sulfate gel electrophoresis. Anal. Biochem. 320, 149–151. 10.1016/s0003-2697(03)00350-6 12895480

[B57] Wilder T. Ryba D. M. Wieczorek D. F. Wolska B. M. Solaro R. J. (2015). N-acetylcysteine reverses diastolic dysfunction and hypertrophy in familial hypertrophic cardiomyopathy. Am. J. Physiol. Heart Circ. Physiol. 309, H1720–H1730. 10.1152/ajpheart.00339.2015 26432840 PMC4666985

[B58] Yotti R. Seidman C. E. Seidman J. G. (2019). Advances in the genetic basis and pathogenesis of sarcomere cardiomyopathies. Annu. Rev. Genomics Hum. Genet. 20, 129–153. 10.1146/annurev-genom-083118-015306 30978303

