# Supplementary material for: Altered coronary artery function, arteriogenesis and endothelial YAP signaling in postnatal hypertrophic cardiomyopathy
Source: Front Physiol. 2023 Mar 31;14:1136852. doi: 10.3389/fphys.2023.1136852 (PMC10102353; doi:10.3389/fphys.2023.1136852)
Supplement: Supplementary file 1 [file Presentation1.pdf]

Supplementary Materials

Altered Coronary Artery Function, Arteriogenesis and Endothelial YAP Signaling in Postnatal Hypertrophic Cardiomyopathy

Paulina Langa[1,3*], Richard J. Marszalek[1,3*], Chad M. Warren[1,3], Shamim K. Chowdhury[1], Monika Halas[1], Ashley Batra[1], Koreena Rafael-Clyke[1], Angelie Bacon[1], Paul H. Goldspink[1,3], R. John Solaro[1,3], Beata M. Wolska[1,2,3,#]
[1]Department of Physiology and Biophysics, College of Medicine, University of Illinois at Chicago
[2]Department of Medicine, Division of Cardiology, College of Medicine, University of Illinois at Chicago
[3]Center for Cardiovascular Research, College of Medicine, University of Illinois at Chicago

*These authors contributed equally and share first authorship
[#]Corresponding author

Beata M. Wolska, PhD

Department of Medicine, Division of Cardiology

University of Illinois at Chicago

840 S Wood St. Rm. 1112 (M/C 715)

Chicago, IL 60612

Ph. 312-413-0240

Fax 312-996-5062

Supplementary Methods

Animal Model

Transgenic (TG) cTnT-R92Q mice were originally generated and characterized in C57Bl/6 genetic background.[1] In our previous[2] and current studies we used cTnT-R92Q mice rederived in FVB/N background (Charles River). All experiments were approved by the Animal Care and Use Committee of the University of Illinois at Chicago and in compliance with the Guide for the Care and Use of Laboratory Animals Eighth Edition as adopted by the U.S. National Institutes of Health. Experiments employed male and female postnatal days 2 (P2), 4 (P4), 7 (P7), 10 (P10), 14 (P14), 21 (P21), and 28 (P28) non-transgenic (NTG) and TG mice expressing an HCM-linked cTnT-R92Q mutant.

Skinned Fiber Bundles Tension Measurement Calculations

Force-Ca^{2+} relation measurements were performed as previously described.[3] Mice were anesthetized with a ketamine/xylazine solution (200mg/20mg/kg body weight). Left ventricular papillary muscles were isolated, dissected into fiber bundles approximately 150-200 µm in width and 3-5 mm in length, and mounted between a micromanipulator and a force transducer. Fiber bundles were detergent-extracted in a high relaxing (HR) solution (10 mmol/L EGTA, 41.89 mmol/L K-Propionate, 100 mmol/L BES, 6.75 mmol/L $MgCl_2$, 6.22 mmol/L Na_2ATP, 10mM Na_2CrP, 5 mmol/L NaN_3, pH 7.0) and with 1% v/v Triton X-100 for 30 min at room temperature. Protease inhibitors (pepstatin A 2.5 µg/ml, leupeptin 1 µg/ml, PMSF 50 µl/ml) and Creatine Phosphokinase (CPK) (1 U/µL) were added to each pCa solution. The sarcomere length was set at 2.2 µm using laser diffraction patterns. The fibers were initially stimulated to generate force at pCa 4.5, and placed back into HR solution. Fibers were then subjected to sequential increases in Ca^{2+} concentration; their developed force was recorded on a chart recorder. At the end of the experiment, the fiber bundles were subjected to pCa 4.5 solutions and the ratio of pre- and post-serial tension measurements were calculated. If the final max force was less than 90% of the original force the fiber was not included in calculations. At the end of the experiments, the width and diameter were measured along three points of fiber length and averaged. The cross-sectional area was

calculated assuming the shape of an ellipse. Force–pCa measurements were calculated by modeling onto a modified Hill equation with nonlinear least-squares regression using the Gauss-Newton algorithm. Tension was calculated by dividing the force by the fiber bundle cross-sectional area.

SDS-PAGE and Immunoblotting

Excised heart samples were immediately frozen in liquid nitrogen and stored at -80°C. Heart samples (10-20mg) were homogenized 1:10 relative to original tissue weight in standard relax buffer (SRB; 75 mM KCl, 10 mM imidazole pH 7.2, 2 mM $MgCl_2$, 2 mM EGTA, 1 mM NaN_3) with protease and phosphatase inhibitors (1:100 v/v Sigma, P-8340, 1:100 v/v Millipore, 524624, and 100 nM of Calyculin A in DMSO Cell Signaling, 9902). The tissue was homogenized at 4°C with a Bead Ruptor 24 Elite (Omni International, 19-040E) at power, 5 m/s; time, 15s; 3 cycles; with a 3 min dwell time between cycles [4, 5]. The homogenized sample was split equally for whole homogenate and myofibril preparations. The myofibril preparations were washed once with Triton X-100 added to 0.5% (v/v) in SRB buffer. The myofibril preparation was spun clarified at 4°C, 15,000 X g for 1 min, and 500µL of SRB with 1% (v/v) Triton X-100 [6] was added to the pellet and incubated on ice with intermittent vortexing for 15 min and this was repeated once. The pellet was washed with SRB without Triton X-100 and resuspended 1:5 relative to the original tissue weight in either industrial strength buffer (ISB: 8M urea, 2M thiourea, 50mM Tris pH 6.8, 3% v/v SDS, 75mM DTT, and 0.05% bromophenol blue[7]) or 2X Laemmli buffer (BioRad, #161-0747). The whole homogenate preparations were solubilized 1:5 relative to original tissue weight in either ISB buffer or 2X Laemmli buffer. The proteins were solubilized by continuous shaking for 15 min at room temperature, sonicated in a water bath for 10 min, and underwent one freeze/thaw cycle. The samples were then heated at 100°C for 3 min and spun clarified at room temperature for 3 min at 21,000 X g, the supernatant was saved as the sample. Protein concentrations were determined with 660 nM Protein Assay (ThermoFisher, 22660) with ICDR reagent. Samples prepared for glutathionylation experiments had minor modifications to the myofibril sample preparation described above. The SRB containing buffers had 25mM NEM [8] added and 2X Laemmli buffer was used to solubilize without

any reducing agents added. The homogenization with the Bead Ruptor 24 Elite (Omni International, 19-040E) used the same parameters except for the number of cycles which was decreased to two.

Whole homogenate protein samples (non-myofilament targets) were loaded (10-25μg/lane) on 12 or 15% (w/v) total acrylamide SDS-PAGE gels, with 0.5% (w/v) bis-acrylamide as previously described [7]. The gels were cast in Bio-Rad's Criterion Cell for most of the experiments except for troponin T, myosin heavy chain, and regulatory light chain (RLC) separations described below. The criterion gels were run in 25mM tris, 192mM glycine, 0.1% (v/v) SDS running buffer at 200v for 1:15 hr:min at room temperature. To effectively separate the TnT variants we adapted a previously described method [9] and loaded myofibril heart samples at 4ug/lane onto 8% (w/v) total acrylamide, with 1.1% (w/v) bis-acrylamide in an SE600 gel box (16cm X 18cm X 1mm thick) (Hoefer). The stacking gel was 4% (w/v) total acrylamide with 3.4% (w/v) bis-acrylamide, and the gel was run at 22mA until the dye front was at the bottom of the gel. Myosin heavy chain isoform separation was carried out in 6% (w/v) total acrylamide SDS-PAGE as previously described [10] with 2ug/lane of myofibril heart sample loaded onto the gel and stained with Coomassie G-250 (Bio-Rad, 1610786) following the manufacture's recommendations. The RLC separations utilized Phos-tag SDS-PAGE as previously described with minor modifications [11] and with 2.5ug/lane of myofibril heart sample loaded onto the gel. RLC was separated into multiple bands corresponding to unphosphorylated (U), one (P1), two (P2), and three (P3) phosphorylation sites; all within the same lane allowing simple ratio analysis. The Phos-tag gel was 12% (w/v) total acrylamide, 3.3% (w/v) bis-acrylamide, 50uM Phos-tag, 100uM MnCl, and poured into 1mm thick Bio-Rad mini gel glass plates. The gel was run in a Bio-Rad mini gel apparatus at 20mA for 75 min at room temperature then the proteins were transferred to the immunoblot membrane.

After the SDS-PAGE was completed, immunoblot transfers were done with the Bio-Rad Criterion Blotter. The protein transfers were done as previously described with some modifications [12]. The protein containing gels were transferred onto 0.2μm polyvinylidene difluoride (PVDF) membrane in 10mM CAPS pH 11.0 without methanol at 20-30V for 90 min chilled with a blue ice pack. The TnT large gel was cut down to a

region of interest and transferred as described above. The transfer of the Phos-tag gels required preincubation with 10mM CAPS pH 11.0, 5mM EDTA for 10 min repeated once and then washed once in 10mM CAPS pH 11.0 buffer before transferring at 30V for 90 min. After the transfer the membranes were blocked with either 5% (w/v) non-fat dry milk (NFDM) in 50mM tris-HCl pH 7.5, 200mM NaCl with 0.1% (v/v) Tween-20 (TBST) or 1-5% BSA-TBST. The glutathionylation blot was blocked with 5% NFDM-TBST with 2.5mM NEM. The immunoblots were incubated in primary antibodies overnight at 4°C, washed in TBST, incubated in secondary antibodies at room temperature for 2hrs, and washed in TBST. See Supplemental table 4 for the specific antibody information. The membranes were developed with ECL (ThermoFisher, 34096 or Bio-Rad, 170-5061), imaged with Chemidoc MP (Bio-Rad), and analyzed with ImageLab (Bio-Rad, v. 6.0.1). The analyzed data were statistically analyzed and graphed with GraphPad Prism v 9.3.1.

To determine overall phosphorylation levels of myofilament proteins, myofibril heart samples (7μg/lane) were loaded onto 15% (w/v) total acrylamide SDS-PAGE cast into empty disposable criterion 26 lane cassettes and run in a Criterion gel box. The gel was stained with Pro-Q Diamond stain (Invitrogen, P33301) following the manufacturer's recommendations. The gel was imaged with Bio-Rad's Chemidoc MP imager, after which, the gel was stained with Coomassie G-250 (Bio-Rad, 1610786) following the manufacturer's recommendations. The images were analyzed with Bio-Rad's Image Lab V 6.0.1 and Microsoft Excel 360. The analyzed data were statistically analyzed and graphed with GraphPad Prism v 9.3.1.

Echocardiography

B-Mode, M-Mode, pulsed-wave Doppler and tissue Doppler images were obtained as previously described[2, 3] from P7, P14 and P28 animals. Mice were anesthetized with an induction of 3-4% isoflurane in an anesthesia chamber followed by maintenance at 1-3% isoflurane concentrations through a respirator. Higher isoflurane concentrations were required in neonatal mice at seven days of age. Body temperature was monitored by a rectal probe and maintained at 37°C. Electrode conduction gel was applied to the distal extremities that were taped onto electrodes. Upper abdominal and

anterior chest wall hair was removed and cleaned away before the application of acoustic conduction gel. The left atrial diameter was assessed by B-mode and M-mode images acquired in the parasternal long-axis window at the level of the aortic root. B-mode and M-mode images were used for multiple parasternal short axis windows (apical, mid-ventricular, and basal) with the mid-ventricular/papillary level singled out for assessment of posterior and anterior wall thickness and ventricular luminal diameter during both systole and diastole to calculate fractional shortening, stroke volume, and cardiac output. The mice were then repositioned to the Trendelenburg position to obtain B-mode and pulse-wave Doppler images of the apical four-chamber window for mitral inflow measurements and tissue Doppler for septal mitral annular velocities. All measurements and calculations were averaged from three consecutive cycles and performed according to the American Society of Echocardiography guidelines. Data analysis was performed with the VevoLab 5.5.1. Analytic Software.

High quality coronary flow velocity signals were obtained from all animals at P7, P14 and P28, under isoflurane induced anesthesia, as described above. The coronary vasodilator properties of isoflurane are well known, so we strictly controlled the level of isoflurane input and heart rate to ensure the accuracy of the collected data. Coronary flow measurements were performed on a modified parasternal long-axis view as previously described[13]. From the low parasternal short-axis view, a search for diastolic color velocity in the anterior interventricular groove followed by clockwise rotation to achieve alignment of the color jet was performed. The sample volume was in a consistent position in all mice during the measurements.

Histology

Mice at 7-, 14- and 28-days postnatal age were anesthetized with 5% isoflurane. Upon absence of pedal reflex, hearts were excised and placed into cold PBS where they were cleaned of extraneous tissue. The hearts were quickly sliced in basal, midpapillary and apical parts, and placed into biopsy cassettes, followed by fixation in 10% neutral buffered formalin (Milipore-Sigma, HT501128), then washed and stored in 70% Ethanol. Next, samples were paraffin embedded, and non-consectuive transverse sectioned were obtained (Research Histology Core, UIC).

The formalin fixed and paraffin embedded slides were deparaffinized with 100% xylene (2 x 7 min) followed by rehydration with incremental washes of decreasing aqueous ethanol (100% for 2 x 5 min, 95% for 5 min, 70% for 5 min, and 50% for 5 min) solutions, and washed in H_2O for 20 min and used for staining.

Fibrosis Assessment

The deparaffinized slides were stained for collagen depositions (fibrosis) using Trichrome Stain kit (Abcam, ab150686) according to manufacture's instructions. The Trichrome stain kit is intended for visualization of collagenous connective tissue fibers in tissue sections. Then slides were mounted with Krystalon toluene-based mounting medium (Harleco, 64969-71). Next, images of whole heart sections were taken by Zeiss Axio Imager Z2 (Germany) brightfield microscope with a motorized stage for tiling. Tiles (region of scanning) were fused using native Zen stitching. Analysis of fibrosis levels in whole heart scans of apex/apical, and midventricular levels were done using ImageJ (NIH ver. 1.53k14) in heart sections harvested at 7, 14, and 28 days postnatally. The Trichrome-stained fibrosis images were analyzed by taking the original RGB image color channels and selecting the color channel corresponding to the trichrome stain. The channel was then manually adjusted to the pixel threshold values that best fit the collagenous staining. The area was measured using ImageJ's Measure tool with the Limit to Threshold property enabled. The tissue/background was determined by minimal auto-thresholding of the same channel. The fraction of collagenous area was calculated by dividing the collagenous area over the tissue area. Localized fibrosis was assessed by 2048 x 2048 square pixel window selection of regions of interest (coronary artery regions – CA, right ventricular insertion – RVI, intraventricular septum- IVS, lateral free wall- LW). Levels of fibrosis were measured as percent collagenous area to tissue area (within the scanned window).

Immunohistochemistry

Antigen retrieval was performed using Tris-EDTA solution at 95° C for 1.5 hours. Slides were then blocked in 5% BSA in TBST (0.1% Tween-20) for 1 hour at room temperature. To visualize vessels, slides were incubated in rat anti-CD31 antibody rabbit anti-YAP) in TBST (Tween 0.1%) overnight at 4°C. Next, after three 5min

washes with TBST (Tween 0.1%), slides were incubated with secondary antibodies (see Supplemental table 4). Slides were washed three times 5min and incubated with DAPI for nuclear counterstaining for 20min at room temperature. Slides were then washed in TBST, and mounted with a mounting medium preserving fluorescent signal (ThermoFisher Scientific, P10144). All slides were tile-scanned (1024 x 1024 pixel size) with z-stacking at 16-bit values and a pinhole diameter of 600.7 µm. Z-stacks were acquired to capture the full depth of the 5µm thick sections followed by maximum-intensity projection. Channels with their properties include:

Age	Gain 1	Gain 2	Gain 3	Gain 4	ILP 1	ILP 2	ILP 3	ILP 4
7	625 V	613 V	663 V	602 V	8.0	0.2	0.2	0.2
14	625 V	613 V	663 V	675 V	6.5	0.2	0.2	0.4
28	645 V	639 V	655 V	496 V	12.0	1.0	0.4	0.4

Dataset-Dependent Confocal Parameters. Channels correspond to 1 – 633 nm, 2 – 561 nm, 3 – 488 nm, 4 – 405 nm. ILP = Illumination Power. 633, Gain: 625.0, Illumination Power(IP): 8.0; 561 Gain: 613.0, IP: 0.2; 488 Gain: 663.0, IP: 0.2; 488 Gain: 602.0, IP: 0.2. Pinhole diameter was maximized (600.721 nm). The resultant images were stitched with ImageJ Grid/Collection Stitching through *.lsm* file metadata.

<u>YAP, αSMA, CD31 Detection and Classification.</u> CD31, α-SMA, and DAPI labeled areas were segmented into masks using Trainable Weka Segmentation (ImageJ plug-in) [14]. To adjust for local signal variation for whole 2D heart images, an adapted classification technique was necessary. Weka supervised machine learning models ("*.model" files) were used to generate the binarized images (masks) for CD31, a-SMA, and DAPI corresponding to endothelial cells, smooth muscle cells, and nuclei, respectively. The models were trained using small cutout sections from tissues of both genotypes at all ages ("*.arff" files). The masks generated from the model were saved in tiff format. Features selected for optimal segmentation included Gaussian blur, Sobel filter, Hessian filter, Difference of Gaussians, and Membrane projections. A membrane thickness of 1, membrane patch size of 19, minimum sigma of 1, and a maximum sigma of 16 were the parameters set for the decision tree. A random forest tree classifier was used for decision-making (I 200-K 2 -S 1025172). Three classes were identified for each

channel, Objects (CD31, a-SMA, or nuclei), background, and autofluorescence. Background masks were generated by first converting the stitched 16-bit color image into an 8-bit grayscale image and running Minimum Autothresholding.
Selections were created from masks. The selections were then superimposed onto the YAP channel for data measurements. Composite selection is a relatively new addition to the ImageJ toolset and is an effective way of analyzing regions without additional image processing steps (multiple background and mask subtractions or multiplications).
The cellular specificity of nuclei was determined by proximity to cell-specific signals – CD31 for endothelial cells and a-SMA for smooth muscle cells. DAPI signal detected near cell-types was expanded to encompass the entire nucleus. To prevent overlap of nuclei classifications (belonging to both groups, endothelial and smooth muscle), nuclei determined from one cell type were subtracted from nuclei of the other. An overlay of different masks and areas analyzed is demonstrated in Supplemental Figure 1.

Arterioles were identified manually by choosing the α-SMA and CD31 segmented objects resembling arterioles, α-SMA circumscribing CD31. The fluorescence intensity signal of YAP was measured in each cell type, differentiating labeling within the nuclei as defined by DAPI staining. Coronary arteries in the basal left ventricular areas were annotated for coronary- and cell-specific YAP detection.

<u>Microvessel Density and Arteriolar Cross-Sectional Area.</u> Microvessel density was measured as the percent coverage of CD31 staining relative to tissue area for whole heart sections and regions of interest. The diameter of the arterioles was approximated by the minor axis of the best fit ellipse. The four main areas of interest - right ventricular insertion (RVI), lateral free wall (LW), peri-coronary (CA), and interventricular septum (IVS) - were annotated in all immunofluorescent images.

Supplementary Table 1. Skinned Fiber Bundles Ca^{2+} Force Measurements.

Variable	2-way ANOVA	7 days NTG	7 days TnT-R92Q	14 days NTG	14 days TnT-R92Q	28 days NTG	28 days TnT-R92Q
**pCa_{50}**	p_g<0.001<br>p_a<0.001<br>p_{ga}=0.002	5.84±0.01	5.97±0.04	5.77±0.02	5.97±0.02	5.80±0.02	6.16±0.05
		p_7 = 0.002<br><br>$p_{NTG7-14}$ = 0.077<br>$p_{NTG7-28}$ = 0.340<br>p_{TG7-14} = 0.837<br>p_{TG7-28} = 0.0003		p_{14} <0.0001<br><br>$p_{NTG7-14}$ = 0.077<br>$p_{NTG14-28}$ =0.573<br>p_{TG7-14} = 0.837<br>$p_{TG14-28}$ <0.0001		p_{28} < 0.0001<br><br>$p_{NTG7-28}$ = 0.340<br>$p_{NTG14-28}$ =0.573<br>p_{TG7-28} = 0.0003<br>$p_{TG14-28}$ <0.0001	
**Hill Coefficient**	p_g <0.001<br>p_a=0.025<br>p_{ga}=0.012	3.40±0.17	2.78±0.21	4.82±0.34	2.71±0.08	4.39±0.27	2.81±0.17
		p_7 = 0.118<br><br>$p_{NTG7-14}$ <0.0001<br>$p_{NTG7-28}$ = 0.009<br>p_{TG7-14} =0.837<br>p_{TG7-28} = 0.953		P_{14} <0.0001<br><br>$p_{NTG7-14}$ <0.0001<br>$p_{NTG14-28}$ =0.009<br>p_{TG7-14} = 0.837<br>$p_{TG14-28}$ = 0.765		P_{28} =0.0003<br><br>$p_{NTG7-28}$ = 0.009<br>$p_{NTG14-28}$ =0.226<br>p_{TG7-28} = 0.953<br>$p_{TG14-28}$ = 0.765	
**Maximum Tension (mN/mm^2)**	p_g=0.006<br>p_a<0.001<br>p_{ga}=0.527	23.86±1.44	18.30±1.87	25.11±1.23	22.90±1.32	30.86±0.57	27.41±1.37
		p_7 = 0.027<br><br>$p_{NTG7-14}$ =0.509<br>$p_{NTG7-28}$ = 0.004<br>p_{TG7-14} =0.049<br>p_{TG7-28} = 0.001		P_{14} =0.192<br><br>$p_{NTG7-14}$ =0.509<br>$p_{NTG14-28}$ =0.004<br>p_{TG7-14} = 0.049<br>$p_{TG14-28}$ =0.037		P_{28} =0.176<br><br>$p_{NTG7-28}$ = 0.004<br>$p_{NTG14-28}$ =0.012<br>p_{TG7-28} =0.001<br>$p_{TG14-28}$ =0.037	
**Sample Size (n)**	NA	8	4	10	13	5	5

Results of Fiber Bundles Analysis. 2-way ANOVA; p_g = genotype; p_a = age; p_{ga} = genotype:age; p_7 = Fisher's LSD test for multiple comparisons between NTG and TnT-R92Q at 7 days of age; p_{14} = P value at 14; p_{28} = P value at 28. pCa_{50} = calcium sensitivity.

Supplementary Table 2. Morphological, Systolic and Diastolic Parameters Evaluated by Echocardiography.

Para-meter	2-way ANOVA	7 days		14 days		28 days	
		NTG	TnT-R92Q	NTG	TnT-R92Q	NTG	TnT-R92Q
LA (mm)	$P_g<0.001$<br>$P_a<0.001$<br>$P_{ga}<0.001$	0.91±0.02	1.00±0.04	1.04±0.03	1.31±0.07	1.52±0.07	2.12±0.06
		$P_7 = 0.2276$<br>$P_{NTG7-14} = 0.067$<br>$P_{NTG7-28} < 0.001$<br>$P_{TG7-14} < 0.001$<br>$P_{TG7-28} < 0.001$		$P_{14} = 0.001$<br>$P_{NTG7-14} = 0.067$<br>$P_{NTG14-28} < 0.001$<br>$P_{TG7-14} < 0.001$<br>$P_{TG14-28} < 0.001$		$P_{28} < 0.001$<br>$P_{NTG7-28} < 0.001$<br>$P_{NTG14-28} < 0.001$<br>$P_{TG7-28} < 0.001$<br>$P_{TG14-28} < 0.001$	
LVIDd (mm)	$P_g=0.490$<br>$P_a<0.001$<br>$P_{ga}=0.354$	2.35±0.07	2.29±0.10	2.71±0.08	2.80±0.11	3.57±0.06	3.40±0.08
		$P_7 = 0.5974$<br>$P_{NTG7-14} = 0.004$<br>$P_{NTG7-28} < 0.001$<br>$P_{TG7-14} < 0.001$<br>$P_{TG7-28} < 0.001$		$P_{14} = 0.4997$<br>$P_{NTG7-14} = 0.004$<br>$P_{NTG14-28} < 0.001$<br>$P_{TG7-14} < 0.001$<br>$P_{TG14-28} < 0.001$		$P_{28} =0.1734$<br>$P_{NTG7-28} < 0.001$<br>$P_{NTG14-28} < 0.001$<br>$P_{TG7-28} < 0.001$<br>$P_{TG14-28} < 0.001$	
LVIDs (mm)	$P_g=0.143$<br>$P_a<0.001$<br>$P_{ga}=0.437$	1.32±0.08	1.27±0.12	1.49±0.10	1.44±0.10	1.96±0.05	1.68±0.12
		$P_7 = 0.7519$<br>$P_{NTG7-14} = 0.217$<br>$P_{NTG7-28} <0.001$<br>$P_{TG7-14} = 0.247$<br>$P_{TG7-28} = 0.004$		$P_{14} = 0.7318$<br>$P_{NTG7-14} = 0.217$<br>$P_{NTG14-28} = 0.003$<br>$P_{TG7-14} = 0.247$<br>$P_{TG14-28} = 0.093$		$P_{28} = 0.0604$<br>$P_{NTG7-28} <0.001$<br>$P_{NTG14-28} = 0.003$<br>$P_{TG7-28} = 0.004$<br>$P_{TG14-28} =0.093$	
RWT (mm)	$P_g=0.525$<br>$P_a=0.542$<br>$P_{ga}=0.795$	0.28±0.01	0.27±0.02	0.28±0.02	0.30±0.01	0.29±0.01	0.30±0.02
		$P_7 = 0.975$<br>$P_{NTG7-14} = 0.915$<br>$P_{NTG7-28} = 0.551$<br>$P_{TG7-14} = 0.312$<br>$P_{TG7-28} = 0.365$		$P_{14} = 0.379$<br>$P_{NTG7-14} = 0.915$<br>$P_{NTG14-28} = 0.622$<br>$P_{TG7-14} = 0.312$<br>$P_{TG14-28} = 0.867$		$P_{28} = 0.825$<br>$P_{NTG7-28} = 0.551$<br>$P_{NTG14-28} = 0.622$<br>$P_{TG7-28} = 0.365$<br>$P_{TG14-28} = 0.867$	
LV Mass (mg)	$P_g =0.466$<br>$P_a<0.001$<br>$P_{ga}=0.027$	11.61±0.55	10.47±0.62	18.03±1.29	21.55±0.97	43.7±2.35	38.63±2.17
		$P_7 = 0.595$<br>$P_{NTG7-14} = 0.915$<br>$P_{NTG7-28} = 0.551$<br>$P_{TG7-14} = 0.312$<br>$P_{TG7-28} = 0.365$		$P_{14} = 0.117$<br>$P_{NTG7-14} = 0.915$<br>$P_{NTG14-28} = 0.622$<br>$P_{TG7-14} = 0.312$<br>$P_{TG14-28} = 0.867$		$P_{28} = 0.21$<br>$P_{NTG7-28} = 0.551$<br>$P_{NTG14-28} = 0.622$<br>$P_{TG7-28} = 0.365$<br>$P_{TG14-28} = 0.867$	

**IVRT (ms)**	$P_g<0.001$<br>$P_a<0.001$<br>$P_{ga}=0.099$	15.20±0.47	19.86±1.25	11.74±0.59	13.85±0.72	10.40±0.41	12.28±0.36
		$P_7 < 0.001$<br>$P_{NTG7-14} = 0.001$<br>$P_{NTG7-28} < 0.001$<br>$P_{TG7-14} < 0.001$<br>$P_{TG7-28} < 0.001$		$P_{14} = 0.044$<br>$P_{NTG7-14} = 0.001$<br>$P_{NTG14-28} < 0.001$<br>$P_{TG7-14} < 0.001$<br>$P_{TG14-28} = 0.120$		$P_{28} = 0.064$<br>$P_{NTG7-28} < 0.001$<br>$P_{NTG14-28} < 0.001$<br>$P_{TG7-28} < 0.001$<br>$P_{TG14-28} = 0.120$	
**E/A Ratio**	$P_g=0.923$<br>$P_a=0.004$<br>$P_{ga}=0.005$	1.22±0.05	1.09±0.06	1.54±0.09	1.33±0.13	1.24±0.03	1.61±0.12
		$P_7 =0.291$<br>$P_{NTG7-14} = 0.001$<br>$P_{NTG7-28} < 0.001$<br>$P_{TG7-14} < 0.001$<br>$P_{TG7-28} < 0.001$		$P_{14} = 0.115$<br>$P_{NTG7-14} = 0.001$<br>$P_{NTG14-28} < 0.001$<br>$P_{TG7-14} < 0.001$<br>$P_{TG14-28} = 0.120$		$P_{28} =0.006$<br>$P_{NTG7-28} < 0.001$<br>$P_{NTG14-28} < 0.001$<br>$P_{TG7-28} < 0.001$<br>$P_{TG14-28} = 0.120$	
**E/e' Ratio**	$P_g=0.006$<br>$P_a=0.161$<br>$P_{ga}=0.039$	39.13±3.00	36.00±5.58	37.35 ±2.11	50.81±4.18	28.99±2.22	45.25±4.29
		$P_7 =0.641$<br>$P_{NTG7-14} = 0.743$<br>$P_{NTG7-28} = 0.077$<br>$P_{TG7-14} = 0.015$<br>$P_{TG7-28} = 0.106$		$P_{14} = 0.020$<br>$P_{NTG7-14} = 0.743$<br>$P_{NTG14-28} = 0.042$<br>$P_{TG7-14} = 0.015$<br>$P_{TG14-28} = 0.312$		$P_{28} = 0.005$<br>$P_{NTG7-28} = 0.077$<br>$P_{NTG14-28} = 0.042$<br>$P_{TG7-28} = 0.106$<br>$P_{TG14-28} = 0.312$	
**ET (ms)**	$P_g<0.001$<br>$P_a=0.001$<br>$P_{ga}=0.981$	51.69±2.58	61.11±4.50	46.63±1.40	56.85±1.38	42.06±1.10	51.42±1.33
		$P_7 = 0.008$<br>$P_{NTG7-14} = 0.142$<br>$P_{NTG7-28} = 0.009$<br>$P_{TG7-14} = 0.230$<br>$P_{TG7-28} = 0.005$		$P_{14} = 0.006$<br>$P_{NTG7-14} = 0.142$<br>$P_{NTG14-28} = 0.199$<br>$P_{TG7-14} = 0.230$<br>$P_{TG14-28} = 0.118$		$P_{28} = 0.009$<br>$P_{NTG7-28} = 0.009$<br>$P_{NTG14-28} = 0.199$<br>$P_{TG7-28} = 0.005$<br>$P_{TG14-28} = 0.118$	
**EF (%)**	$P_g=0.031$<br>$P_a=0.442$<br>$P_{ga}=0.260$	76.60±2.06	76.77±3.65	75.92±2.11	80.54±1.85	75.77±0.50	83.40±1.90
		$P_7 = 0.957$<br>$P_{NTG7-14} = 0.832$<br>$P_{NTG7-28} = 0.802$<br>$P_{TG7-14} = 0.257$<br>$P_{TG7-28} = 0.037$		$P_{14} = 0.166$<br>$P_{NTG7-14} = 0.832$<br>$P_{NTG14-28} = 0.963$<br>$P_{TG7-14} = 0.257$<br>$P_{TG14-28} = 0.376$		$P_{28} =0.022$<br>$P_{NTG7-28} = 0.802$<br>$P_{NTG14-28} = 0.963$<br>$P_{TG7-28} = 0.037$<br>$P_{TG14-28} = 0.376$	

**HR (bmp)**	P_g=0.013<br>P_a=0.063<br>P_{ga}=0.939	488.0±17.29	444.4±30.97	478.7±23.27	440.9±20.05	533.6±18.16	480.8±12.47
		P_7 = 0.147<br>$P_{NTG7-14}$ = 0.754<br>$P_{NTG7-28}$ = 0.144<br>P_{TG7-14} = 0.909<br>P_{TG7-28} = 0.211		P_{14} = 0.223<br>$P_{NTG7-14}$ = 0.754<br>$P_{NTG14-28}$ = 0.080<br>P_{TG7-14} = 0.909<br>$P_{TG14-28}$ = 0.187		P_{28} = 0.083<br>$P_{NTG7-28}$ = 0.144<br>$P_{NTG14-28}$ = 0.080<br>P_{TG7-28} = 0.211<br>$P_{TG14-28}$ = 0.187	
**SV (µl)**	P_g>0.999<br>P_a<0.001<br>P_{ga}=0.419	14.63±1.02	13.71±0.84	21.55±1.09	23.69±1.67	42.44±1.39	41.21±1.86
		P_7 = 0.637<br>$P_{NTG7-14}$ < 0.001<br>$P_{NTG7-28}$ < 0.001<br>P_{TG7-14} < 0.001<br>P_{TG7-28} < 0.001		P_{14} = 0.289<br>$P_{NTG7-14}$ < 0.001<br>$P_{NTG14-28}$ < 0.001<br>P_{TG7-14} < 0.001<br>$P_{TG14-28}$ < 0.001		P_{28} = 0.530<br>$P_{NTG7-28}$ < 0.001<br>$P_{NTG14-28}$ < 0.001<br>P_{TG7-28} < 0.001<br>$P_{TG14-28}$<0.001	
**CO (ml/min)**	P_g=0.021<br>P_a<0.001<br>P_{ga}=0.080	7.23±0.66	5.94±0.26	10.39±0.88	10.53±1.02	23.49±0.31	19.83±1.07
		P_7 = 0.266<br>$P_{NTG7-14}$ = 0.007<br>$P_{NTG7-28}$ < 0.001<br>P_{TG7-14} < 0.001<br>P_{TG7-28} < 0.001		P_{14} = 0.909<br>$P_{NTG7-14}$ = 0.007<br>$P_{NTG14-28}$ < 0.001<br>P_{TG7-14} < 0.001<br>$P_{TG14-28}$ < 0.001		P_{28} = 0.003<br>$P_{NTG7-28}$ < 0.001<br>$P_{NTG14-28}$ < 0.001<br>P_{TG7-28} < 0.001<br>$P_{TG14-28}$ < 0.001	
**N**		8	8	8	7	7	9

Results for Echocardiography measurements Analysis.Data presented as mean ± SEM. 2-way ANOVA; P_g = genotype; P_a = age; P_{ga} = genotype:age. Fisher's LSD test was used for multiple comparisons between NTG and TnT=R92Q at 7 (P_7), 14 (P_{14}) and 28 (P_{28}) days of age. LA = left atrium, LVIDd = left ventricular internal diameter at diastole, LVIDs – left ventricular internal diameter at systole, RWT = relative wall thickness, LV = left ventricle, IVRT = isovolumic relaxation time, E = peak velocity of early diastolic transmitral flow, A = peak velocity of late diastolic transmitral flow, e' – peak velocity of early diastolic mitral annular motion, ET = ejection time, EF = ejection fraction, HR = heart rate, SV = stroke volume, CO = cardiac output, N = Sample sizes.

Supplementary Table 3. Coronary flow measurement parameters.

Para-meter	2-way ANOVA	7 days		14 days		28 days	
		NTG	TnT-R92Q	NTG	TnT-R92Q	NTG	TnT-R92Q
**Mean Systolic Velocity (mm/s)**		116.3±12.65	58.45±4.01	51.14±7.91	74.28±7.74	97.38± 7.95	92.07±10.95
	p_g=0.090<br>p_a=0.004<br>p_{ga}<0.001	P_7 < 0.001<br><br>$P_{NTG7-14}$<0.001<br>$P_{NTG7-28}$=0.166<br>P_{TG7-14}=0.261<br>P_{TG7-28}=0.014		P_{14} = 0.093<br><br>$P_{NTG7-14}$<0.001<br>$P_{NTG14-28}$=0.001<br>P_{TG7-14}=0.261<br>$P_{TG14-28}$=0.181		P_{28} = 0.687<br><br>$P_{NTG7-28}$=0.166<br>$P_{NTG14-28}$=0.001<br>P_{TG7-28}=0.014<br>$P_{TG14-28}$=0.181	
**N**		8	7	8	7	7	9
**Peak Systolic Velocity (mm/s)**		185.3±20.18	91.31±5.84	77.31±11.17	111.3±11.35	153.4±13.40	141.1±16.71
	p_g=0.049<br>p_a=0.002<br>p_{ga}<0.001	P_7 < 0.001<br><br>$P_{NTG7-14}$<0.001<br>$P_{NTG7-28}$=0.133<br>P_{TG7-14}=0.357<br>P_{TG7-28}=0.018		P_{14} = 0.109<br><br>$P_{NTG7-14}$<0.001<br>$P_{NTG14-28}$<0.001<br>P_{TG7-14}=0.357<br>$P_{TG14-28}$=0.148		P_{28} = 0.545<br><br>$P_{NTG7-28}$=0.133<br>$P_{NTG14-28}$<0.001<br>P_{TG7-28}=0.018<br>$P_{TG14-28}$=0.148	
**N**		8	7	8	7	7	9
**Mean Diastolic Velocity (mm/s)**		338.7±28.07	222.5±18.81	216.8±29.88	241.1±28.59	369.4±21.40	314.6± 9.83
	p_g =0.017<br>p_a<0.001<br>p_{ga}=0.018	P_7 = 0.001<br><br>$P_{NTG7-14}$<0.001<br>$P_{NTG7-28}$=0.391<br>P_{TG7-14}=0.585<br>P_{TG7-28}=0.008		P_{14} = 0.478<br><br>$P_{NTG7-14}$<0.001<br>$P_{NTG14-28}$<0.001<br>P_{TG7-14}=0.585<br>$P_{TG14-28}$=0.037		P_{28} = 0.129<br><br>$P_{NTG7-28}$=0.391<br>$P_{NTG14-28}$<0.001<br>P_{TG7-28}=0.008<br>$P_{TG14-28}$=0.037	
**N**		8	8	8	7	6	8
**Peak Diastolic Velocity (mm/s)**		561.0±46.59	366.5±31.88	359.4±47.94	404.0±48.18	607.4±34.88	534.6±17.68
	p_g=0.028<br>p_a<0.001<br>p_{ga}=0.015	P_7 < 0.001<br><br>$P_{NTG7-14}$<0.001<br>$P_{NTG7-28}$=0.432<br>P_{TG7-14}=0.508<br>P_{TG7-28}=0.004		P_{14} = 0.431<br><br>$P_{NTG7-14}$<0.001<br>$P_{NTG14-28}$<0.001<br>P_{TG7-14}=0.508<br>$P_{TG14-28}$=0.025		P_{28} = 0.221<br><br>$P_{NTG7-28}$=0.432<br>$P_{NTG14-28}$<0.001<br>P_{TG7-28}=0.508<br>$P_{TG14-28}$=0.025	

**N**		8	8	8	7	6	8
**Coronary Diastolic Accelera-tion Time (ms)**	p_g<0.001<br>p_a<0.001<br>p_{ga}=0.884	21.91 ± 0.69	28.74 ± 1.87	17.82 ± 1.17	24.13 ± 1.04	17.28 ± 1.71	22.84 ± 0.98
		P_7 < 0.001<br>$P_{NTG7-14}$=0.029<br>$P_{NTG7-28}$=0.017<br>P_{TG7-14}=0.018<br>P_{TG7-28}=0.002		P_{14} = 0.002<br>$P_{NTG7-14}$=0.029<br>$P_{NTG14-28}$=0.771<br>P_{TG7-14}=0.018<br>$P_{TG14-28}$=0.002		P_{28} = 0.004<br>$P_{NTG7-28}$=0.017<br>$P_{NTG14-28}$=0.771<br>P_{TG7-28}=0.002<br>$P_{TG14-28}$=0.002	
**N**		8	8	8	7	7	9
**Flow time/ Cardiac Cycle**	p_g=0.004<br>p_a=0.073<br>p_{ga}=0.313	0.54 ± 0.02	0.51 ± 0.02	0.60 ± 0.02	0.50 ± 0.02	0.59 ± 0.03	0.55 ± 0.01
		P_7 = 0.352<br>$P_{NTG7-14}$=0.056<br>$P_{NTG7-28}$=0.080<br>P_{TG7-14}=0.903<br>P_{TG7-28}=0.132		P_{14} = 0.005<br>$P_{NTG7-14}$=0.056<br>$P_{NTG14-28}$=0.917<br>P_{TG7-14}=0.903<br>$P_{TG14-28}$=0.116		P_{28} = 0.129<br>$P_{NTG7-28}$=0.080<br>$P_{NTG14-28}$=0.917<br>P_{TG7-28}=0.132<br>$P_{TG14-28}$=0.116	
**N**		8	8	8	7	7	9

Results for Coronary flow Doppler measurements Analysis. Data presented as mean ± SEM. 2-way ANOVA; P_g = genotype; P_a = age; P_{ga} = genotype:age. Fisher's LSD test was used for multiple comparisons between NTG and TnT=R92Q at 7 (P_7), 14 (P_{14}) and 28 (P_{28}) days of age.

Supplementary Table 4. Antibodies for Western blot, immunofluorescence and immunohistochemical staining

Target Antibodies	Cat. number	Supplier	Dilution
Rb YAP	14074S	Cell Signaling Technology	1:1000; 5% NFDM + TBST
Rb Phospho-YAP Ser127	4911	Cell Signaling Technology	1:1000; 2% BSA + TBST
Rb GSK3α/β	5676	Cell Signaling Technology	1:1000; 2% BSA + TBST
Rb Phospho- GSK3α/β	8566S	Cell Signaling Technology	1:1000; 2% BSA + TBST
Rb Calcineurin	PA5-17446	Thermo Fisher Scientific	1:1000; 5% NFDM + TBST
Rb PKA-C	5842S	Cell Signaling Technology	1:1000; 2% BSA + TBST
Rb Phospho-PKA-C	5661S	Cell Signaling Technology	1:1000; 2% BSA + TBST
Rb ERK1/ERK2	9102	Cell Signaling Technology	1:1000; 2% BSA + TBST
Rb Phospho-ERK1/ERK2	76299	Abcam	1:2000; 2% BSA + TBST
Ms PLN	A010-14	Badrilla	1:2000; 5% NFDM + TBST
Rb Phospho-PLN Ser16	07-052	EMD Millipore	1:1000; 5% NFDM + TBST
Rb Phospho-PLN Thr17	A010-13AP	Badrilla	1:2500; 5% NFDM + TBST
Ms CAMKII	sc5306	Santa Cruz	1:200; 5% NFDM + TBST
Rb Phospho-CAMKII	PA5-37833	Invitrogen	1:1000; 2% BSA + TBST
Rb SERCA2a	A010-23	Badrilla	1:5000; 5% NFDM + TBST
Ms eNOS	AB76198	Abcam	1:1000; 5% NFDM + TBST
Rb Gp91phox NOX2	Ab12968	Abcam	1:3000; 5% NFDM + TBST
Rb NOX4	GR309720-5	Abcam	1:2000; 5% NFDM + TBST
Ms Actin	10R-7820	Fitzgerald	1:2000; 5% NFDM + TBST
Rb Actin	PA1-16889	ThermoFisher	1:10000; 5% NFDM + TBST
Hs-anti-mouse-HRP 2°antibody	7076S	Cell Signaling Technology	1:25,000; 5% NFDM + TBST
Gt-anti-mouse-HRP 2°antibody	7074S	Cell Signaling Technology	1:20,000; 5% NFDM + TBST
Ms Troponin I	10R-T123K	Fitzgerald Industries International	1:5000; 5% NFDM + TBST
Ms Glutathione	101-A-250	Virogen	1:1000; 5% NFDM + TBST
Ms Troponin T	MS-295-PO	ThermoFisher	1:1000; 1% BSA + TBST
Rb Phospho-troponin I (S23/24)	4004S	Cell Signaling Technology	1:1000; 1% BSA + TBST
Rb MyBP-C	custom	Gift from Rick Moss	1:10000; 5% NFDM + TBST

Ms RLC	ALX-BC-1150-S-L001	Enzo Life Sciences	1:2000; 5% NFDM + TBST
Rt CD31	DIA310	Dianova	1:10; 1%BSA + TBST
Rb YAP	14074S	Cell signaling Technology	1:100; 1%BSA + TBST
Ms α-SMA	AB7817	Abcam	1:100; 1%BSA + TBST
Gt-anti-rat Alexa Fluor633	A21094	ThermoFisher Scientific	1:1000; 1%BSA + TBST
Gt-anti-rabbit Alexa Fluor568	A11011	ThermoFisher Scientific	1:1000; 1%BSA + TBST
Cicken anti-mouse Alexa Fluor 488	A21206	ThermoFisher Scientific	1:1000; 1%BSA + TBST

Abbreviations used: Chicken, chicken antibody; Ms, mouse antibody; Rt, rat antibody; Rb, rabbit antibody; Hs, horse antibody; Gt, goat antibody: NFDM, non-fat dry milk; TBST, Tris-buffered saline with 0.1% (v/v) Tween-20; HRP, horseradish peroxidase; BSA, bovine serum albumin.

References

1. Tardiff JC, Hewett TE, Palmer BM, Olsson C, Factor SM, Moore RL, Robbins J, Leinwand LA. Cardiac troponin T mutations result in allele-specific phenotypes in a mouse model for hypertrophic cardiomyopathy. *The Journal of clinical investigation* 1999;**104**:469-481.
2. Chowdhury SAK, Warren CM, Simon JN, Ryba DM, Batra A, Varga P, Kranias EG, Tardiff JC, Solaro RJ, Wolska BM. Modifications of Sarcoplasmic Reticulum Function Prevent Progression of Sarcomere-Linked Hypertrophic Cardiomyopathy Despite a Persistent Increase in Myofilament Calcium Response. *Front Physiol* 2020;**11**:107.
3. Alves ML, Dias FAL, Gaffin RD, Simon JN, Montminy EM, Biesiadecki BJ, Hinken AC, Warren CM, Utter MS, Davis RTR, Sakthivel S, Robbins J, Wieczorek DF, Solaro RJ, Wolska BM. Desensitization of myofilaments to Ca2+ as a therapeutic target for hypertrophic cardiomyopathy with mutations in thin filament proteins. *Circulation Cardiovascular genetics* 2014;**7**:132-143.
4. Batra A, Warren CM, Ke Y, McCann M, Halas M, Capote AE, Liew CW, Solaro RJ, Rosas PC. Deletion of P21-activated kinase-1 induces age-dependent increased visceral adiposity and cardiac dysfunction in female mice. *Mol Cell Biochem* 2021;**476**:1337-1349.
5. Capote AE, Batra A, Warren CM, Chowdhury SAK, Wolska BM, Solaro RJ, Rosas PC. B-arrestin-2 Signaling Is Important to Preserve Cardiac Function During Aging. *Front Physiol* 2021;**12**:696852.
6. Solaro RJ, Pang DC, Briggs FN. The purification of cardiac myofibrils with Triton X-100. *Biochim Biophys Acta* 1971;**245**:259-262.
7. Fritz JD, Swartz DR, Greaser ML. Factors affecting polyacrylamide gel electrophoresis and electroblotting of high-molecular-weight myofibrillar proteins. *Anal Biochem* 1989;**180**:205-210.
8. Hill BG, Ramana KV, Cai J, Bhatnagar A, Srivastava SK. Measurement and identification of S-glutathiolated proteins. *Methods in enzymology* 2010;**473**:179-197.
9. Anderson PA, Malouf NN, Oakeley AE, Pagani ED, Allen PD. Troponin T isoform

expression in humans. A comparison among normal and failing adult heart, fetal heart, and adult and fetal skeletal muscle. *Circ Res* 1991;**69**:1226-1233.

10. Warren CM, Greaser ML. Method for cardiac myosin heavy chain separation by sodium dodecyl sulfate gel electrophoresis. *Anal Biochem* 2003;**320**:149-151.

11. Kinoshita E, Kinoshita-Kikuta E, Takiyama K, Koike T. Phosphate-binding tag, a new tool to visualize phosphorylated proteins. *Mol Cell Proteomics* 2006;**5**:749-757.

12. Matsudaira P. Sequence from picomole quantities of proteins electroblotted onto polyvinylidene difluoride membranes. *J Biol Chem* 1987;**262**:10035-10038.

13. Chang WT, Fisch S, Chen M, Qiu Y, Cheng S, Liao R. Ultrasound based assessment of coronary artery flow and coronary flow reserve using the pressure overload model in mice. *J Vis Exp* 2015:e52598.

14. Arganda-Carreras I, Kaynig V, Rueden C, Eliceiri KW, Schindelin J, Cardona A, Sebastian Seung H. Trainable Weka Segmentation: a machine learning tool for microscopy pixel classification. *Bioinformatics* 2017;**33**:2424-2426.

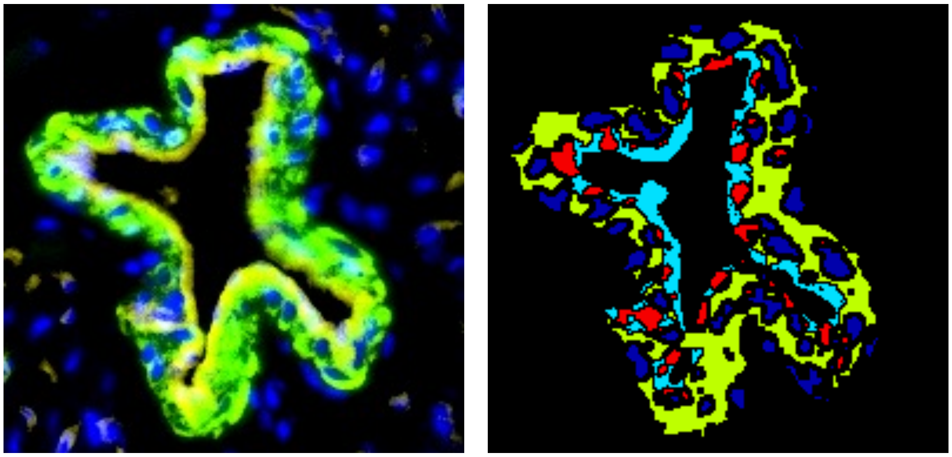

Supplementary Figure 1. Original image of CD31 (yellow), α-SMA (green), and DAPI (blue) on the left. Masks were generated from scripts on the right with endothelial and smooth muscle cells represented by teal and green whereas their nuclei were represented by red and dark blue respectively.

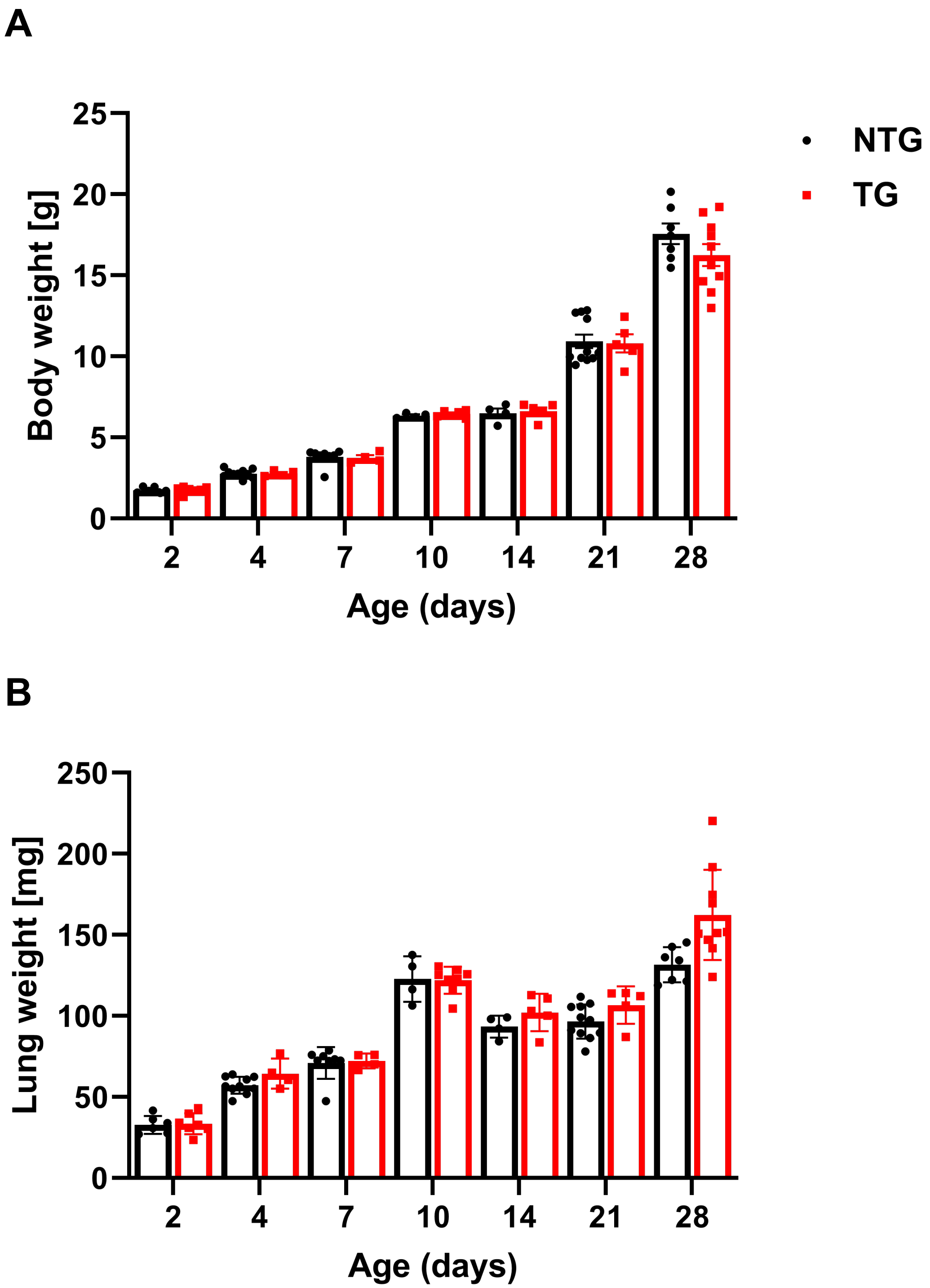

Supplementary Figure 2. Age-dependent change in body and lung weights. (A) Body weight and **(B)** lung weight in NTG and TG mice. Data presented as mean ± SEM and analyzed using Two-way ANOVA followed by Fisher's LSD test; NTG N=4-10,TG N=4-10.

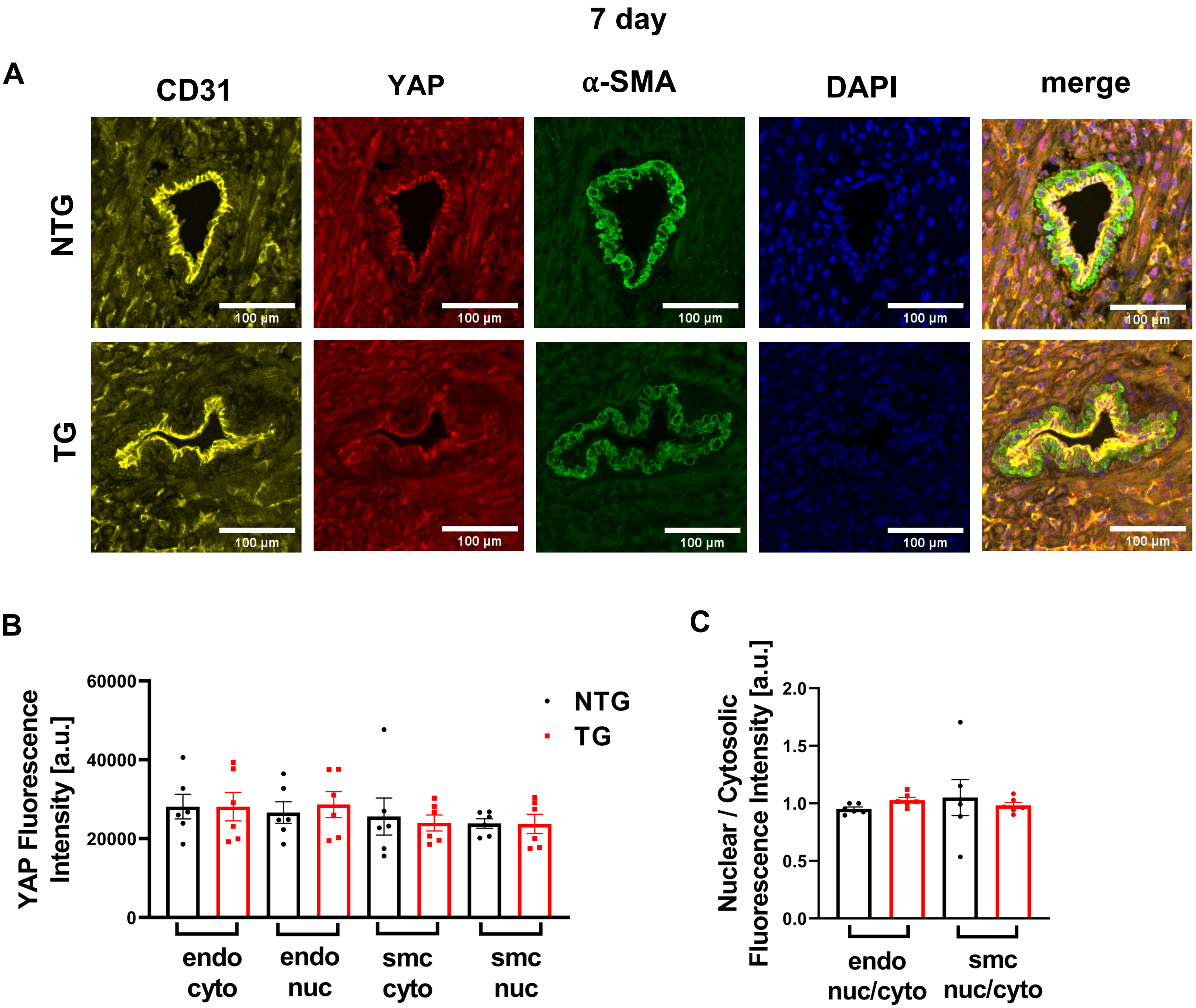

Supplementary Figure 3. Expression of YAP at P7. (A) Representative immunohistochemistry (IHC) images of coronary vessels from NTG and TH hearts stained with fluorescent antibodies against CD31, YAP, α-SMA and DAPI. The merge images represent CD31/YAP/α-SMA with nuclear DAPI counterstained coronary artery region at the basal section of the heart at P7. **(B)** Cytosolic and nuclear YAP fluorescence signal in endothelial (endo) and smooth muscle cells (smc) in the heart sections and **(C)** a ratio of nuclear/cytosolic YAP expression in the endothelial and smooth muscle cells (smc) in the heart sections. Data presented as mean ± SEM and analyzed using One-way ANOVA followed by Fisher's LSD. NTG N=6, TG N=4-6. NTG, non-transgenic; TG, transgenic.

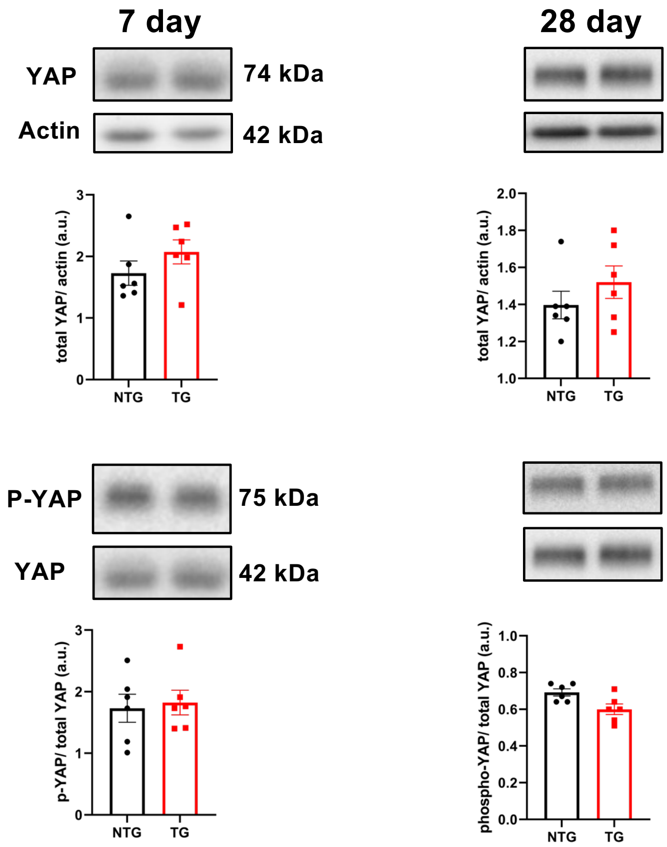

Supplementary Figure 4. Time-dependent expression and localization of YAP. Western blot analysis of YAP abundance (upper panel) and phosphorylation (p-YAP) (Ser-127) (lower panel) at P7 and P28 in TG and NTG hearts. Data presented as mean ± SEM and analyzed using Student's t-test NTG N=6, TG N=6. NTG, non-transgenic; TG, transgenic.

Supplementary Figure 5

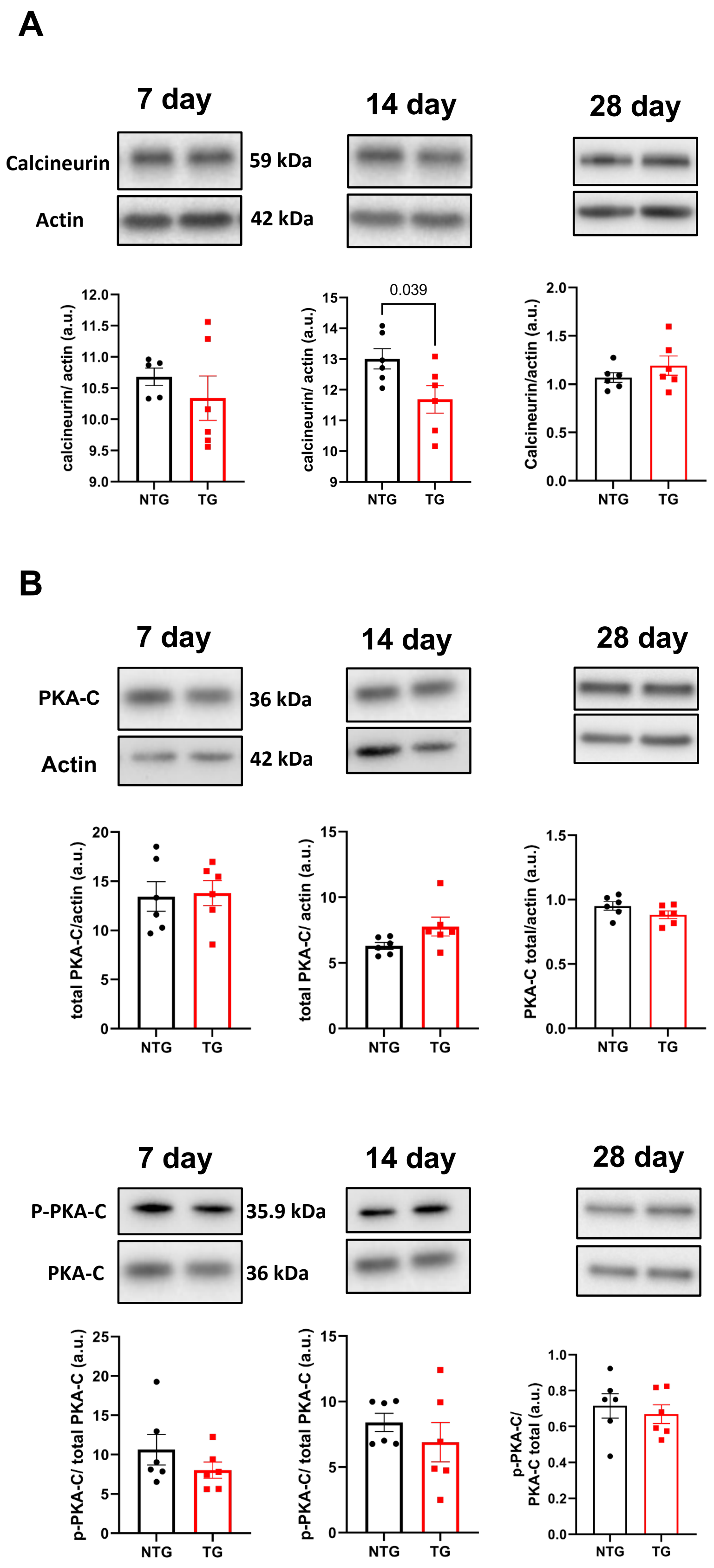

Supplementary Figure 5. Time-dependent changes in expression and phosphorylation of Calcineurin and PKA-C. (A) Western blot analysis of Calcineurin abundance. **(B)** Western blot analysis of PKA-C abundance and phosphorylation of PKA-C (Thr197). NTG, non-transgenic; TG, transgenic. Data presented as mean ± SEM and analyzed using unpaired Student's t-test, NTG N= 6, TG N=5-6.

Supplementary Figure 6

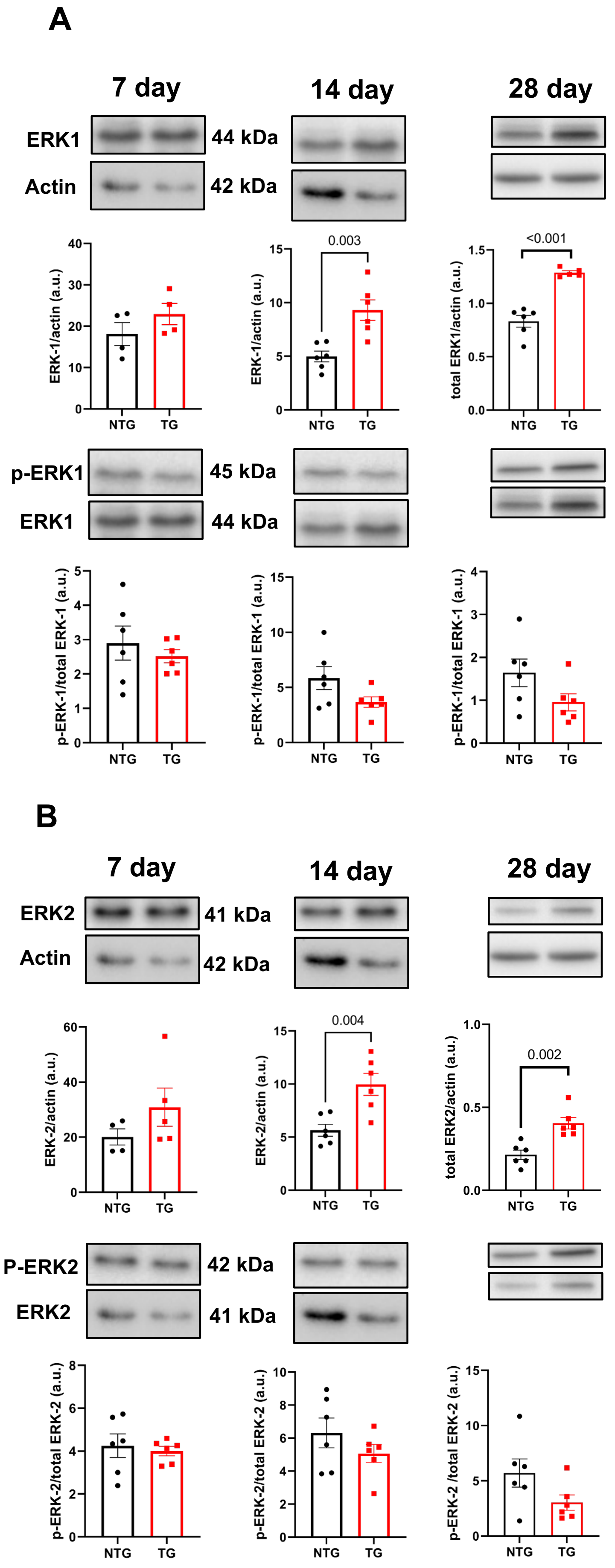

Supplementary Figure 6. **Time-dependent changes in expression and phosphorylation of ERK1/2. (A)** Western blot analysis of ERK1 abundance and phosphorylation (Thr202 and Tyr204) of ERK1 and **(B)** Western blot analysis of ERK2 abundance and phosphorylation (Thr185 and Tyr187) of ERK2. NTG, non-transgenic; TG, transgenic. Data presented as mean ± SEM and analyzed using unpaired Student's t-test, NTG N= 6, TG N=6.

Supplementary Figure 7

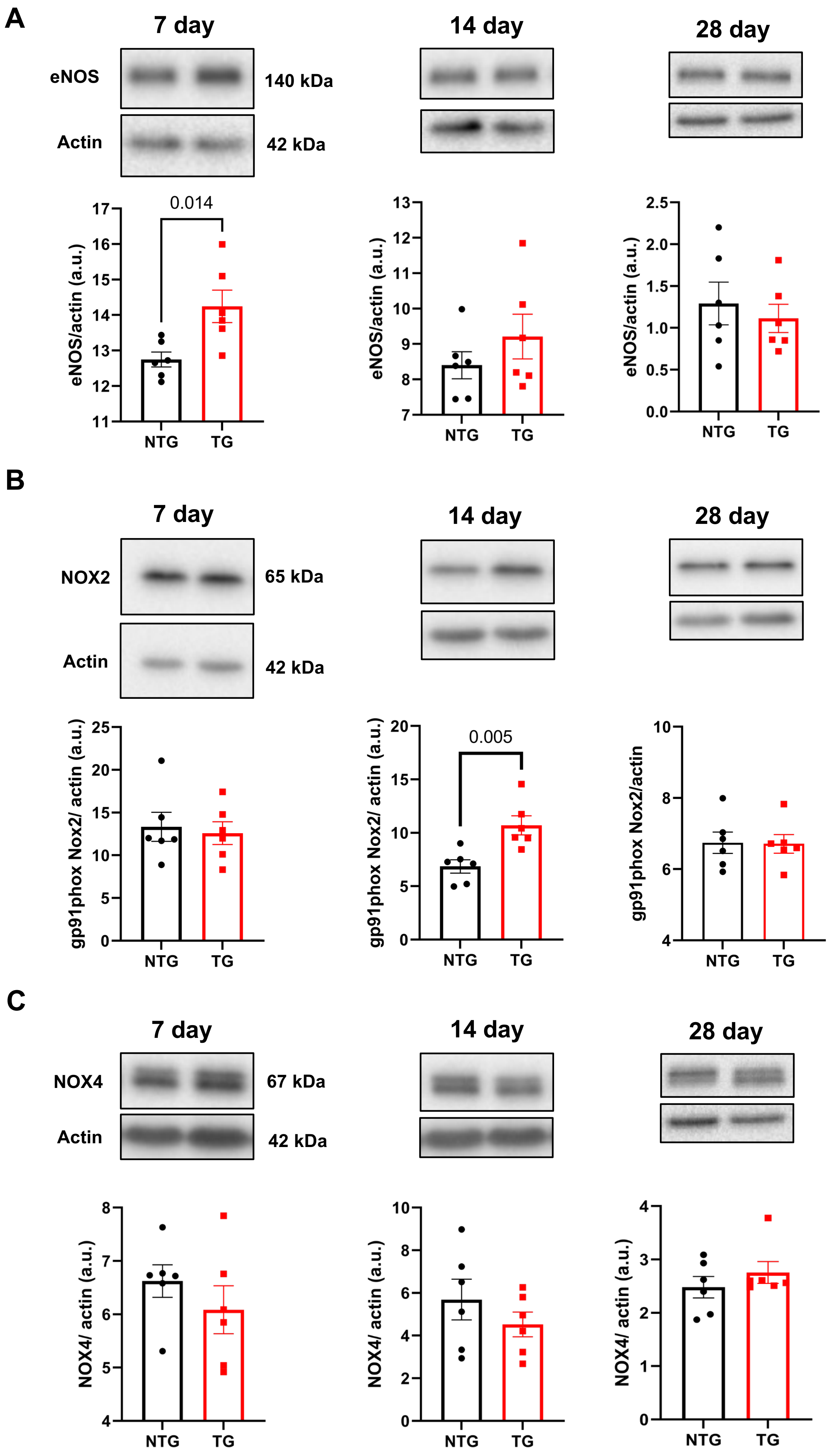

Supplementary Figure 7. The effects of R92Q-cTnT and Age on ROS-associated signaling. **(A)** Representative Western blots and summary data of eNOS, **(B)** Gp91phox-NOX2, and **(C)** NOX4 in NTG and TG hearts. Data are presented as mean ± SEM and analyzed using unpaired Student's t-test. NTG N=5-6, TG n=6.

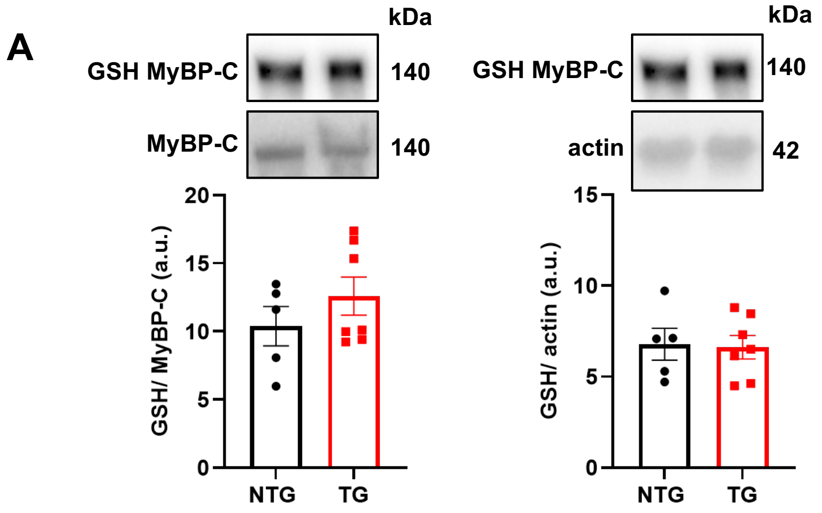

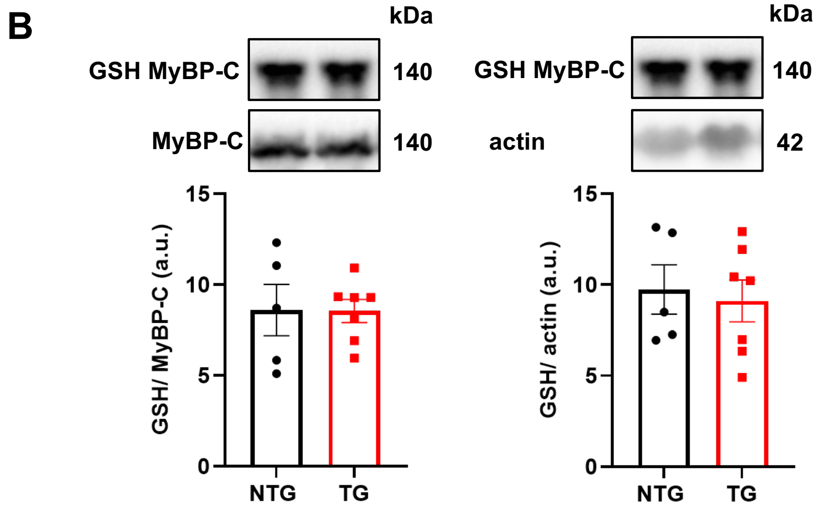

Supplementary Figure 8. The effects of R92Q-cTnT and age on glutathionylation (GSH). (A) Western blot analysis of glutathione in P7 hearts. The left panel is the analysis of glutathionylation/ myosin binding protein-C and the right panel is the analysis of glutathionylation/actin. Representative Western blot images are above the histograms. **(B)** Western blot analysis of glutathione in P14 hearts. The left panel is the analysis of glutathionylation/ myosin binding protein-C and the right panel is the analysis of glutathionylation/actin. Data are presented as mean ± SEM and analyzed using unpaired Student's t-test N=5-7.

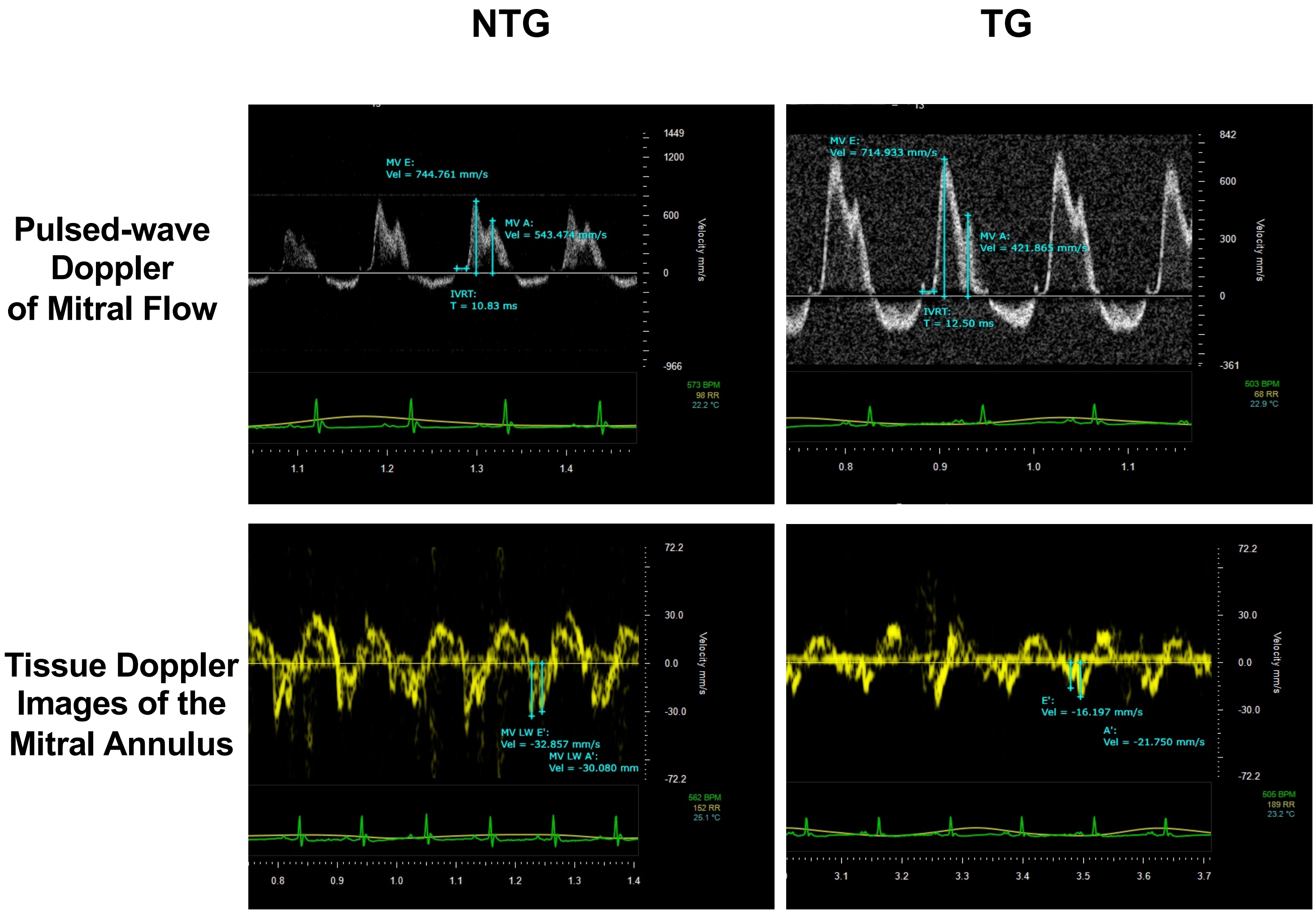

Supplementary Figure 9. Representative recordings of pulsed Doppler of mitral inflow (top) and tissue Doppler images of the mitral annulus (bottom) at P28. E – peak velocity of early diastolic transmitral flow, A - peak velocity of late diastolic transmitral flow, e' – peak velocity of early diastolic mitral annular motion, IVRT = isovolumic relaxation time.

Supplemental Figure 10

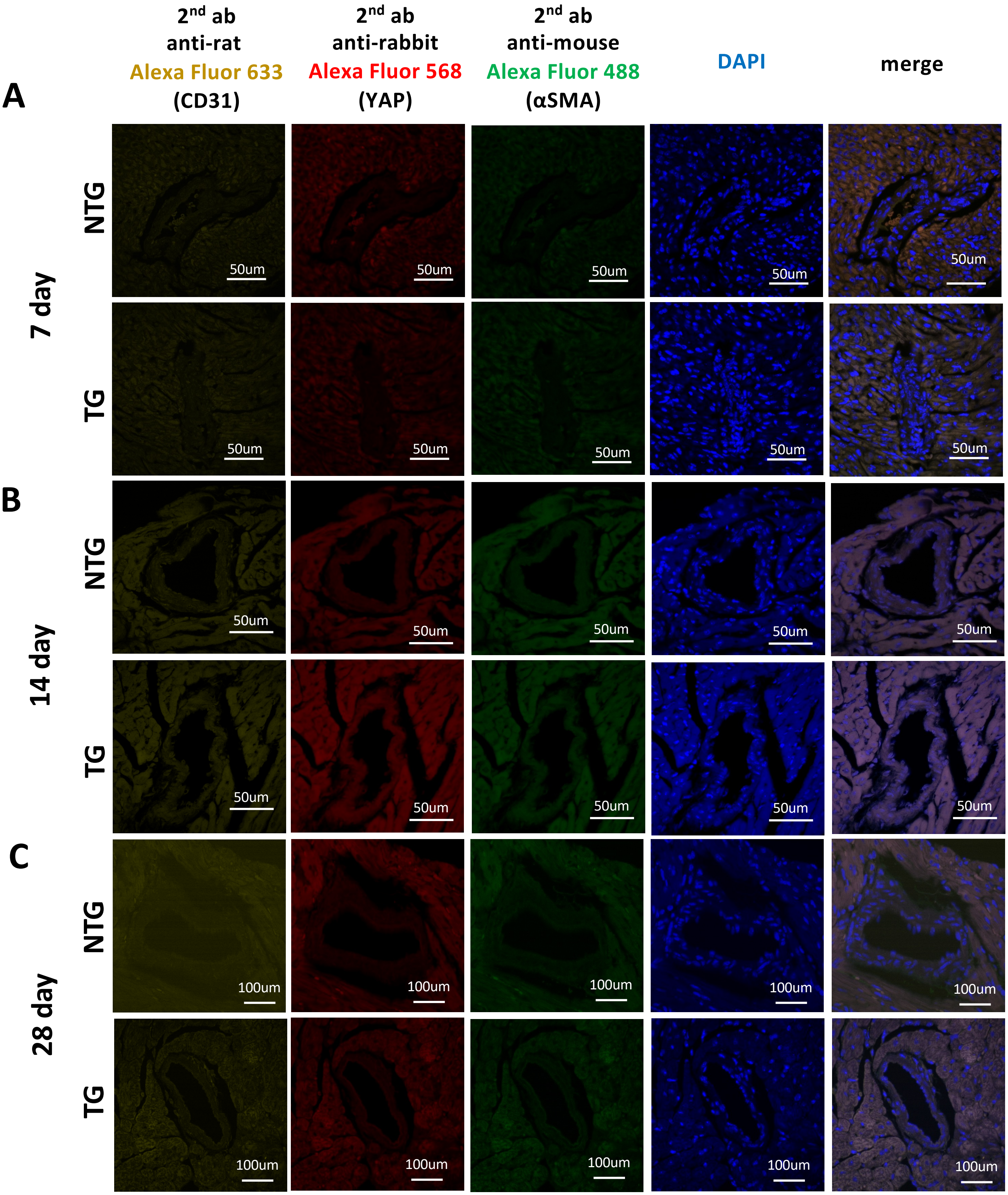

Supplementary Figure 10. Representative immunohistochemistry (IHC) images of coronary vessels from NTG and TG hearts stained with fluorescent secondary antibodies (no primary antibody). The columns show (from left to right): goat anti-rat Alexa Fluor 633 (used to visualize CD31 staining shown in Figure 8), goat anti-rabbit Alexa Fluor 568 (used to visualize YAP staining shown in Figure 8), and chicken anti-mouse Alexa Fluor 488 (used to visualize αSMA staining shown in Figure 8), DAPI nuclear staining, and merged channels. The color for each antibody was chosen for efficient visualization and does not represent the actual color of the fluorescent dye conjugated with each secondary antibody. Panel (A) shows representative images of NTG and TG coronary vessel specimens harvested at 7 days of age; (B) at 14 days of age; and (C) at 28 days of age.
